# Rare tandem repeat expansions associate with genes involved in synaptic and neuronal signaling functions in schizophrenia

**DOI:** 10.1038/s41380-022-01857-4

**Published:** 2022-11-16

**Authors:** Jia Wen, Brett Trost, Worrawat Engchuan, Matthew Halvorsen, Linda M. Pallotto, Aleksandra Mitina, NaEshia Ancalade, Martilias Farrell, Ian Backstrom, Keyi Guo, Giovanna Pellecchia, Bhooma Thiruvahindrapuram, Paola Giusti-Rodriguez, Jonathan David Rosen, Yun Li, Hyejung Won, Patrik K. E. Magnusson, Ulf Gyllensten, Anne S. Bassett, Christina M. Hultman, Patrick F. Sullivan, Ryan K. C. Yuen, Jin P. Szatkiewicz

**Affiliations:** 1 Department of Genetics, University of North Carolina, Chapel Hill, NC 27599 USA; 2 Genetics and Genome Biology, The Hospital for Sick Children, Toronto, ON M5G 1X8 Canada; 3 The Centre for Applied Genomics, The Hospital for Sick Children, Toronto, ON M5G 1X8 Canada; 4 Department of Psychiatry, University of Florida College of Medicine, Gainesville, FL 32610 USA; 5 Department of Biostatistics, University of North Carolina, Chapel Hill, NC 27599 USA; 6 Department of Medical Epidemiology and Biostatistics, Karolinska Institutet, Stockholm, 17177 Sweden; 7 Department of Immunology, Genetics and Pathology, Science for Life Laboratory, Uppsala University, Uppsala, 75185 Sweden; 8 Clinical Genetics Research Program and Campbell Family Mental Health Research Institute, Centre for Addiction and Mental Health, Toronto, ON M6J 1H4 Canada; 9 The Dalglish Family 22q Clinic for Adults with 22q11.2 Deletion Syndrome, Toronto General Hospital, and Toronto General Hospital Research Institute, University Health Network, Toronto, ON M5G 2C4 Canada; 10 Department of Psychiatry, University of Toronto, Toronto, ON M5S 1A8 Canada; 11 Department of Psychiatry, University of North Carolina, Chapel Hill, NC 27599 USA; 12 Department of Molecular Genetics, University of Toronto, Toronto, ON M5S 1A8 Canada

**Keywords:** Genetics, Psychiatric disorders

## Abstract

Tandem repeat expansions (TREs) are associated with over 60 monogenic disorders and have recently been implicated in complex disorders such as cancer and autism spectrum disorder. The role of TREs in schizophrenia is now emerging. In this study, we have performed a genome-wide investigation of TREs in schizophrenia. Using genome sequence data from 1154 Swedish schizophrenia cases and 934 ancestry-matched population controls, we have detected genome-wide rare (<0.1% population frequency) TREs that have motifs with a length of 2–20 base pairs. We find that the proportion of individuals carrying rare TREs is significantly higher in the schizophrenia group. There is a significantly higher burden of rare TREs in schizophrenia cases than in controls in genic regions, particularly in postsynaptic genes, in genes overlapping brain expression quantitative trait loci, and in brain-expressed genes that are differentially expressed between schizophrenia cases and controls. We demonstrate that TRE-associated genes are more constrained and primarily impact synaptic and neuronal signaling functions. These results have been replicated in an independent Canadian sample that consisted of 252 schizophrenia cases of European ancestry and 222 ancestry-matched controls. Our results support the involvement of rare TREs in schizophrenia etiology.

## Introduction

Schizophrenia is a chronic and debilitating mental disorder. Twin, family, and adoption studies consistently support a genetic basis for schizophrenia, with estimates of heritability in the range of 60-65% from pedigree data, and around 81% from twin data [1–3]. Tremendous progress has been made toward the identification of genetic variants that confer schizophrenia risk, including 270 loci harboring common variants, 8 large rare copy number variants, and 10 genes implicated from exome sequencing studies of rare coding variants [4–6]. Nonetheless, the genetic variants identified thus far for schizophrenia confer less risk than its heritability estimates [4–6]. Tandem repeats are a plausible source for some of this missing heritability [4, 7, 8]; however, until recently tandem repeats were difficult to interrogate due to the technical difficulty of resolving complex variants in repetitive regions from short-read sequencing [4] and in genotyping/imputing tandem repeats using biallelic SNP arrays.

Tandem repeats occur in DNA where sequences of one or more nucleotides are repeated directly adjacent to each other. They are a major source of genetic variation in the human genome [9, 10]. The repetitive sequence in a tandem repeat can cause DNA slippage during DNA replication or repair, leading to increases in repeat size across generations. Pathogenic tandem repeat expansions (TREs) are currently known to cause over 60 disorders, most of which affect the central nervous system [7, 11–13]. TREs can alter both coding and non-coding regions of genes and exert harmful effects via a variety of pathophysiological mechanisms (reviewed in [7, 12]). TREs in the coding sequence can result in abnormally long stretches of polyglutamine or polyalanine, leading to protein misfolding and aggregation (e.g., expanded CAG repeats in *HTT* in Huntington’s disease). TREs in the noncoding sequence can occur in 5’ or 3’ untranslated regions (UTRs), introns, promoters, or enhancers. The impact of noncoding TREs depends on the type, length, and locations of the repeats, which may include epigenetic gene silencing, RNA toxicity mediated by protein titration, repeat-associated non-AUG translation, modulation of enhancer activity, and disruptions of boundaries demarcating 3D chromatin domains [7, 12–15].

Due to their repetitiveness and abundance in the human genome, tandem repeats are challenging to study at a genome-wide scale [7]. Recent studies by Trost et al. and Mitra et al. developed computational methods to evaluate genome-wide tandem repeats in autism spectrum disorder (ASD) using data generated from short-read whole genome sequencing (WGS), and together they provide compelling evidence that TREs contribute to ASD susceptibility [16, 17]. For schizophrenia, genome-wide evaluations of tandem repeats are now emerging [18]. Published WGS studies examined TREs in a number of specific loci known to be associated with monogenic neurological diseases and found several of these variants in schizophrenia patients [19, 20]. In an attempt to identify causative variants underlying *CACNA1C*, one of the loci being replicated in multiple common variant associations for schizophrenia and bipolar disorder, Song et al. found that an intronic tandem repeat was associated with a higher risk of developing psychiatric disorders and decreased enhancer activity [21]. Most recently, Mojarad et al applied methods developed in ASD studies and evaluated genome-wide tandem repeats in 252 schizophrenia cases and 222 controls [18]. Despite their relatively small sample size, the results from Mojarad et al suggest that rare TREs are an important class of variants contributing to the etiology of schizophrenia. These examples, as well as the known genetic overlap between schizophrenia and ASD [22, 23], highlight the need for further systematic investigations of TREs in schizophrenia.

In this study, we carry out a genome-wide investigation in the role of rare TREs in schizophrenia etiology. We first apply a genome-wide TRE detection pipeline [16] to identify TREs from WGS data in a Swedish sample of schizophrenia cases and controls. We then assess the possible functional effects of these variants by comparing burdens of rare TREs between cases and controls in genic and intergenic regions, in different parts of genes, in gene-sets previously identified to increase risk for schizophrenia or neurodevelopmental disorders, as well as in conserved sequences and epigenomic annotations empirically derived from the human brain. Finally, we replicate significant associations in an independent Canadian cohort [18]. Our results suggest that rare TREs collectively contribute to the genetic risk of schizophrenia.

## Materials and methods

### Subject recruitment and ethics approval

We have complied with all relevant ethical regulations. The study protocol and all procedures on data from human research subjects were approved by the appropriate ethical committees in Sweden and the United States (Karolinska Institutet [Regionala Etikprövningsnämnden, Stockholm], University of Uppsala [Regionala Etikprövningsnämnden, Uppsala], and University of North Carolina Institutional Review Boards). All participants gave written informed consent.

The primary WGS dataset used in this study consists of 1159 Swedish schizophrenia cases and 936 ancestry-matched population control individuals [19]. Full descriptions of the cohort are available elsewhere [19] and are briefly summarized here. The schizophrenia cases were selected from the Swedish Schizophrenia Study [24] to have typical Swedish ancestry, unequivocal schizophrenia case status (>8 inpatient or outpatient psychiatric treatment contacts for schizophrenia or schizoaffective disorder, ≥30 inpatient days for schizophrenia, ≥5 redeemed prescriptions for antipsychotics, and few or no treatment contacts for bipolar disorder), and without any known pathogenic CNVs (e.g., 22q11.2 deletion). Carriers of known pathogenic CNVs were previously identified from SNP array genotyping [25] and were not included in this study because here we aim to evaluate the contribution of novel loci to schizophrenia risk. Controls were group matched to cases by ancestry and were selected from the SweGen project (unrelated individuals originating from the Swedish Twin Registry [26]). We also included WGS data derived from 2504 unrelated samples from the phase three panel of the 1000 Genomes Project (1000GP [27]). The 1000GP cohort included individuals from 26 populations, representing five continental regions of the world.

### Whole genome sequencing data

Individuals from the schizophrenia case-control cohort were previously sequenced by our group at the National Genomics Infrastructure platform in Sweden [19]. DNA was extracted from blood. DNA libraries were prepared from ~1 μg DNA using Illumina TruSeq PCR-free DNA sample preparation kits targeting an insert size of 350 bp in accordance with the manufacturer’s instructions. Libraries were sequenced on the Illumina HiSeq X platform using 2 ×150 base pair (bp) cycles (2 × 150 bp paired-end reads) to a target depth of 30x (minimum 21x, median 37x). The 1000GP sample collection was sequenced by The New York Genome Center [27]. DNA was extracted from lymphoblastoid cell lines. DNA libraries were prepared from 1 μg DNA using the Illumina TruSeq DNA PCR-free (450 bp) Library Preparation Kit in accordance with the manufacturer’s instructions. Libraries were sequenced on an Illumina NovaSeq 6000 sequencer using 2 ×150 bp cycles to a target depth of 30x (minimum 27x, mean 34x).

### Genome-wide detection of tandem repeats, TREs, and rare TREs

We used ExpansionHunter Denovo (EHdn) [28], an efficient catalog-free method for genome-wide tandem repeat detection from short-read WGS data. We applied the density-based spatial clustering of applications with noise (DBSCAN) algorithm to identify TREs whose lengths were outliers compared with other members of the cohort [16, 29]. We defined rare TREs as TREs that were found in less than 0.1% of the 1000GP population controls. All genomic coordinates are given in NCBI Build 38/UCSC hg38. A detailed description of the detection methods is provided in Supplementary Methods.

### Statistical analyses

A detailed description of quality control, genome annotations and statistical analyses is provided in Supplementary Methods.

### Gel electrophoresis

Selected tandem repeats predicted by EHdn were validated with gel electrophoresis size separation of PCR products of the regions of interest. The following coordinates (hg38) were amplified using the reagents, primers. and conditions specified. Takara PrimeSTAR GXL DNA Polymerase (GXL) and Qiagen HotStarTaq DNA Polymerase (HotStar) kits were used. General thermocycling conditions are as follows: GXL - 1 min 98 °C, 37x (10 s 98 °C, 15 s variable, 1 min 30 s 68 °C), 10 min 68 °C. HotStar – 15 min 95 °C, 37x (30 s 95 °C, 30 s variable, 1 min 15 s 72 °C), 10 min 72 °C. Target specific information: *PDIA5* chr3:123151603-123152369, F-GCCTTCATAGCAGACATAAGCC, R - TCTGCCAGAGGTTGAGTCAC, HotStar with Q solution, Anneal 63 °C; *GABRA1* chr5:161663263- 161663867, F - GCAAGAAAGGGGAGTTACCG, R - CCTAACACCTCATGCTGTACC, GXL, Anneal 60 °C. The sample NA12878 available from Coriell was used as the reference control. A 100 bp ladder was used for size reference (FroggaBio 100 bp).

### Replication

We obtained replication association results from an independent dataset from Canada that included 252 unrelated adult cases with schizophrenia of European ancestry and 222 ancestry-matched individuals with no major neuropsychiatric disorders (after removal of 8 samples being outliers of genome-wide tandem repeat count) [18]. This dataset is well-suited for replication because the sequencing technology and TRE detection methods used for the replication samples were identical to those used for our Swedish case-control samples. Replication was attempted for all significant association results in the Swedish case-control comparisons. For each attempted region, we performed burden testing of rare TREs in cases versus controls in the replication samples and obtained association summary statistics. We then used METAL [30] to perform a fixed-effect meta-analysis using the inverse-variance-based method to merge the findings between the original and the replication studies. Details of the replication samples, TRE detection, quality control and statistical analyses are documented in Supplementary Methods.

## Results

**Fig. 1:**
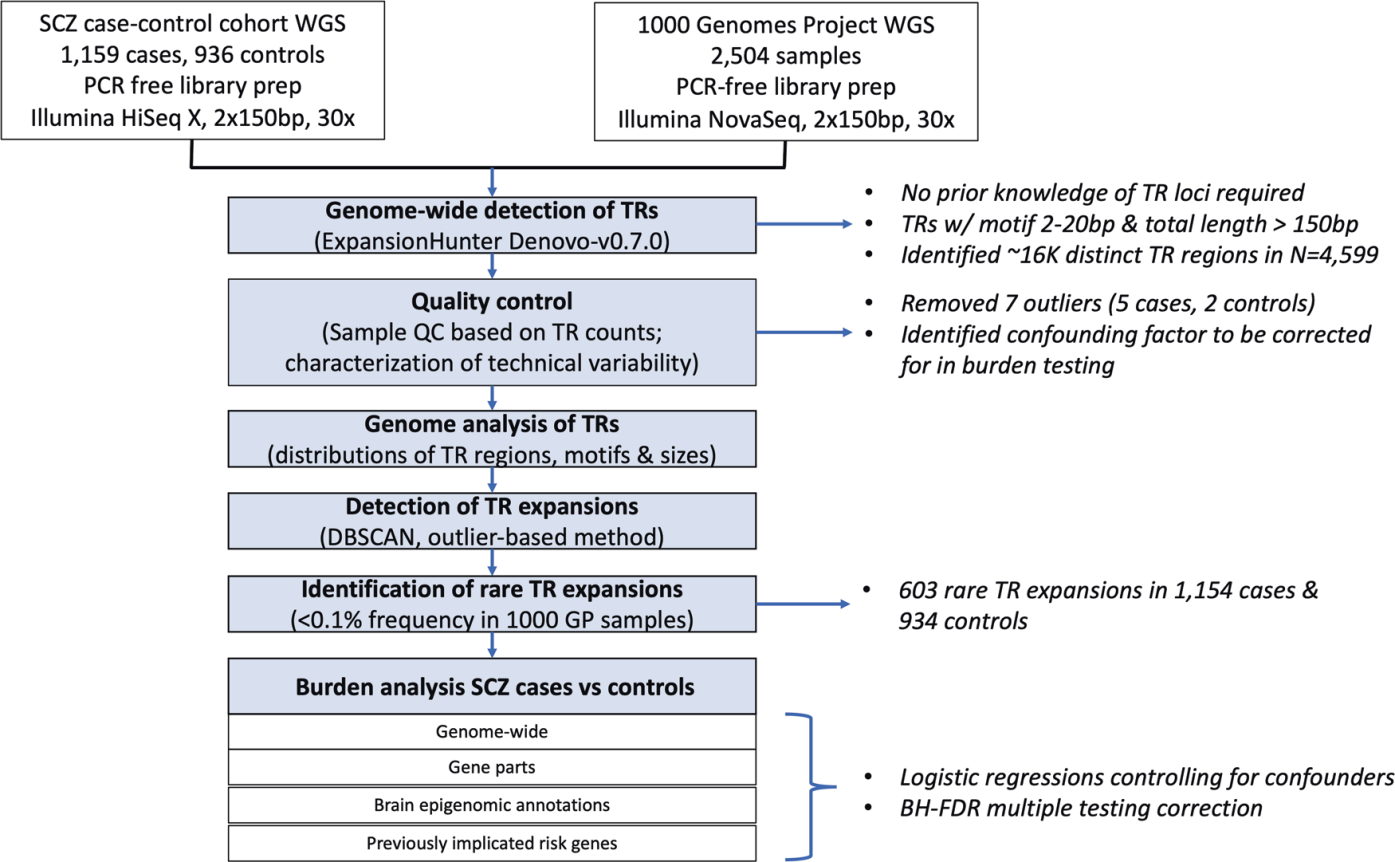
Study overview. This flowchart summarizes the study design and analytic workflow.

We used WGS data from 1159 schizophrenia cases and 936 ancestry-matched population controls from Sweden that were previously generated by our group [19] (Fig. 1). To estimate population frequency of tandem repeats, we included WGS data for 2504 genomes sequenced by the 1000 GP [27]. All WGS data were sequenced on Illumina platforms using 150 bp paired-end reads to a similar mean coverage per sample.

### Identification of novel tandem repeats expanded in schizophrenia

Using EHdn [28] we performed genome-wide detection of tandem repeats that have motifs between 2 and 20 bp and total length greater than the read length (150 bp) across all the samples. Seven samples (5 schizophrenia cases and 2 controls) were removed because they were outliers in terms of genome-wide tandem repeat count (Supplementary Fig. 1), resulting in 4592 genomes for subsequent analyses (1154 schizophrenia cases, 934 controls, and 2504 samples from 1000GP).

We defined a tandem repeat-containing region as a genomic location containing tandem repeats with one or more different motifs overlapping by at least 1 bp. Across all 4592 samples, we identified 21,153 unique tandem repeat motifs in 16,723 distinct regions (Supplementary Table 1). The technical characteristics of our tandem repeat data, including the distributions of motif, motif size, and tandem-repeat-containing regions, were consistent with those reported previously [16, 18] (Supplementary Figs. 2–4). Tandem-repeat-containing regions were enriched in GC-rich regions (odds ratio [OR] = 1.04, P < 2.2e-16) but depleted in conserved DNA sequences defined by phyloP [31] (OR = 0.015, P < 2.2e-16) and phastCons [32] (OR = 0.208, P < 2.2e-16, Supplementary Table 2, Supplementary Fig. 5). We compared our data to the known simple sequence repeat regions in the human reference genome and to tandem repeat loci reported in ASD [16]. Of the 16,723 tandem-repeat-containing regions reported here, 3447 (20.6%) of them have not been previously reported (Supplementary Fig. 6).

Pathogenic TREs are typically significantly longer than what is observed in the general population. For example, patients with fragile X syndrome typically carry >200 CGG repeats in the 5’ UTR of *FMR1*, while unaffected individuals generally have 6 to 53 repeats. Following Trost et al. [16], we defined a TRE as a tandem repeat that is much larger than most other members of the study cohort. We applied DBSCAN [29], a non-parametric clustering algorithm, to tandem repeat calls across the 4592 post-QC samples. Outliers for repeat length of each tandem repeat motif were deemed to be TREs. A total of 2890 TREs were identified, and 1559 (53.9%) of those were novel in comparison to the TREs reported in Trost et al. [16].

We deemed TREs in the schizophrenia case-control cohort to be rare when found in less than 0.1% of the 1000 GP samples. This resulted in 603 rare TREs for subsequent burden testing (Supplementary Table 3). We examined the distribution of the count of rare TREs per sample using a stratified histogram (Supplementary Fig. 7). We did not observe any samples that were outliers based on rare TRE count (Supplementary Fig. 7).

### Contribution of rare tandem repeat expansions in schizophrenia

To assess the possible functional effects of rare TREs, we used burden testing to evaluate whether rare TREs are enriched in different genomic annotations in schizophrenia cases versus controls. Only autosomal TREs were retained for burden analysis. Our power calculation suggested that we had ≥80% power to detect association signals with burden testing when the aggregated minor allele frequency was 0.01 (i.e. aggregated minor allele count of 20), the genotypic relative risk was ≥4.9, and assuming a type I error level of 1×10^−5^ (Supplementary Fig. 8). Burden testing was performed using logistic regression models that allowed us to correct for confounding factors that may cause spurious association signals as described in Supplementary Methods. To identify potential confounding variables, we carried out a principal component (PC) analysis of the normalized anchored in-repeat-read counts which suggested the inclusion of PC2, PC3, and PC8 as covariates in the logistic regression models (Supplementary Fig. 9 and Supplementary Table 4). After correcting for these PCs along with sex, we did not find evidence of inflation based on the estimated effect measured by the burden of rare TREs in intergenic regions (see below *Genome-wide burden*), which is consistent with a prior report [16]. Furthermore, for burden testing in target regions (i.e. global genic regions, gene parts, gene sets, epigenomic annotations), we additionally included global intergenic burden as a covariate in the logistic regression models to correct for any confounding factors that may have not been accounted for by the PC2, PC3, and PC8, and to increase the specificity of the tests in target regions.

#### Genome-wide burden

**Fig. 2:**
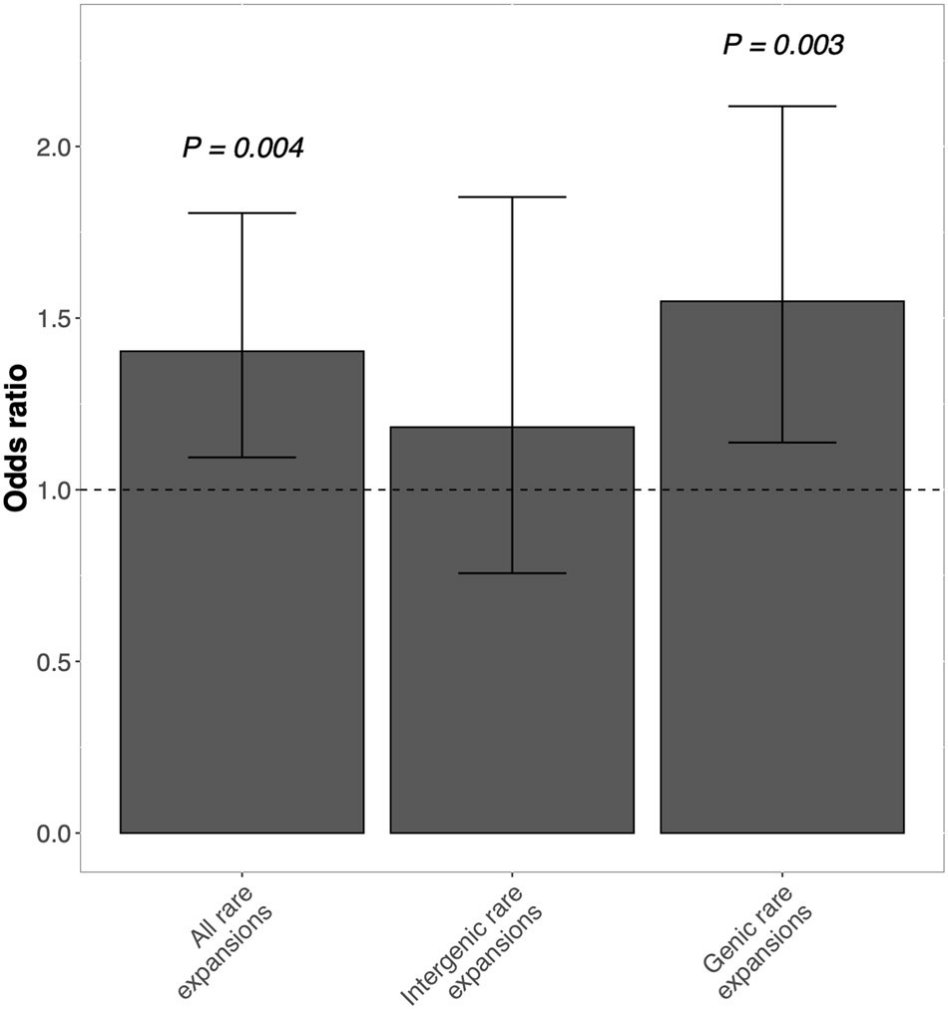
Genome-wide burden of rare TREs in schizophrenia. The error bars denote 95% confidence interval.

We first compared the total number of rare TREs in schizophrenia cases versus controls in three ways: genome-wide, in genic regions only, and in intergenic regions only. When a rare TRE overlapped with any gene/transcript by at least 1 bp, it was defined as genic; otherwise, it was intergenic. We observed a significantly increased burden in schizophrenia for rare TREs (OR = 1.403, P = 0.004) and genic rare TREs (OR = 1.549, P = 0.003; Fig. 2). No statistically significant difference between cases and controls was detected for rare TREs in intergenic regions (OR = 1.182, P = 0.232; Fig. 2). To understand how repeat motifs may influence case-control burden comparison, we stratified genic TREs into CpG-containing (any rare genic TRE that contains the sequence of CG, GC, CGC, CGG, CCG, GCC, GGC or GCG) or non-CpG-containing, and performed burden testing for each group separately. There were 249 CpG-containing rare genic TREs and 694 non-CpG-containing rare genic TREs across 1,154 schizophrenia and 934 control samples. The burdens of both CpG-containing and non-CpG-containing genic rare TREs were significantly increased in schizophrenia cases compared to controls. (Supplementary Table 5).

Given our finding that tandem repeats were depleted in conserved DNA sequences, we next compared the total conserved base pairs affected by rare TREs in cases versus controls and observed a modestly elevated burden in schizophrenia (OR = 1.005, P = 0.021; Supplementary Table 6). We further performed burden analysis for coding conserved base pairs and non-coding conserved base pairs separately and found that the elevated burden is significantly contributed by conserved base pairs in non-coding regions (Supplementary Table 6).

We estimated the proportion of samples carrying rare TREs using the residuals of rare TRE counts after controlling for confounding factors. Using this approach, the estimated sample proportions were 11.35% in schizophrenia cases and 4.39% in controls, i.e., a 6.96% excess in schizophrenia (Wilcoxon ranked sum test P = 8.83e-9).

#### Burden in different parts of genes

**Fig. 3:**
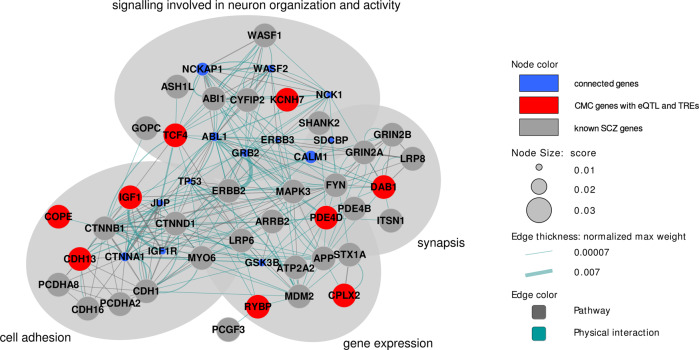
Network of genes with rare TREs that are differentially expressed in schizophrenia. Interactions between genes were extracted from GeneMANIA. Top 50 scoring connected genes were pulled from pathways or physical interactions from the network data with automatic weighting. Node color: gray for known schizophrenia genes (defined in Methods), red for eQTL genes with rare TREs that are differentially expressed in schizophrenia (CMC genes with eQTLs and rare TREs); and blue for additional connected genes (top 50 genes) pulled from GeneMANIA. Node size is proportional to the strength of predictions for a given gene function.

Previous studies found that, in ASD, rare exonic TREs and rare TREs affecting splicing were enriched, while *de novo* tandem repeat mutations were enriched in brain regulatory regions [16, 17]. Motivated by these examples, we first compared the total number of rare TREs in schizophrenia cases versus controls in different parts of protein coding genes (Fig. 3 and Supplementary Table 7). Interestingly, we found a higher burden of rare intronic (OR = 1.436, P = 0.030) and rare splicing TREs (OR = 2.174, P = 0.024), although the excess was not statistically significant after multiple testing correction (BH- corrected P = 0.105 for both).

#### Burden in brain epigenomic annotations

We then compared the total number of rare TREs in schizophrenia cases versus controls within functional annotations experimentally derived from human brain tissue known to affect gene expression (Supplementary Table 8). These annotations include open chromatin regions from ATAC-seq [33], chromatin binding factor CTCF from ENCODE [34], boundaries of topologically associating domains (TADs) [35] including level 1 sub-TAD boundaries and level 2 sub-TAD boundaries, differential neuronal cell specific histone modifications (H3K27ac and H3K4me3) peaks [36], neuronal frequently interacting regions (FIRE, superFIRE) [37], and neuronal chromatin interactions [37]. The bin size was 40 kb for sub-TADs and FIREs, 10 kb for enhancer-promoter annotations, and for the remaining annotations ranged from 0.126 kb to 880 kb. We leveraged neuronal annotations when available as previous work has indicated neurons as the central cell type harboring genetic risk for schizophrenia [5, 36–38]. We observe a higher burden of rare TREs in sub-TAD boundaries – level 1 (OR = 4.977, P = 0.012), sub-TAD boundaries – level 2 (OR = 4.476, P = 0.022), and enhancer-promoter anchors (OR = 1.506, P = 0.026) in schizophrenia cases, but they were not statistically significant after multiple testing correction (BH-corrected *P* = 0.105, Supplementary Table 8).

#### Burden in gene sets previously implicated in schizophrenia and neurodevelopmental disorders

In fragile X syndrome, both abnormally expanded CGG repeats and point mutations in *FMR1* have been reported [39]. Motivated by this example, we hypothesized that schizophrenia-associated TREs may be enriched in genes known to increase risk for schizophrenia, previously identified via common variant association [5], copy number variation [4], exome sequencing [6], or gene expression studies [40, 41]. Given the known genetic overlap between schizophrenia and ASD [22, 23], we also included risk genes previously implicated in neurodevelopmental disorders via exome sequencing [42–44], copy number variation [45–47] or tandem repeats studies [6]. Burden testing compared the total number of rare TREs in cases versus controls within each of the 21 gene sets considered (Methods, Supplementary Table 9). We found an excess of rare TREs in schizophrenia cases in brain-expressed genes that are differentially expressed (DEGs) between schizophrenia and controls as determined by the Common Mind Consortium (i.e. CMC brain DEGs; OR = 6.63, P = 0.005, BH-corrected P = 0.063), the genes with expression quantitative trait loci (eQTLs) in human brain as identified by PsychENCODE Integrative Analysis (OR = 1.73, P = 2.14e-3, BH-corrected P = 0.063), as well as in the SynGO ontology category postsynapse process (OR = 27.94, P = 0.004, BH-corrected P = 0.063). These are further supported by the fact that many of the CMC brain DEGs with rare TREs and the genes with brain eQTLs overlapping with CMC brain DEGs are highly connected with other known schizophrenia genes and they are involved in similar functions in synaptic or neuronal signaling (Fig. 3). While examining specific genes within the three significant gene sets, we found that the rare TREs mostly affected introns (Supplementary Table 10).

**Fig. 4:**
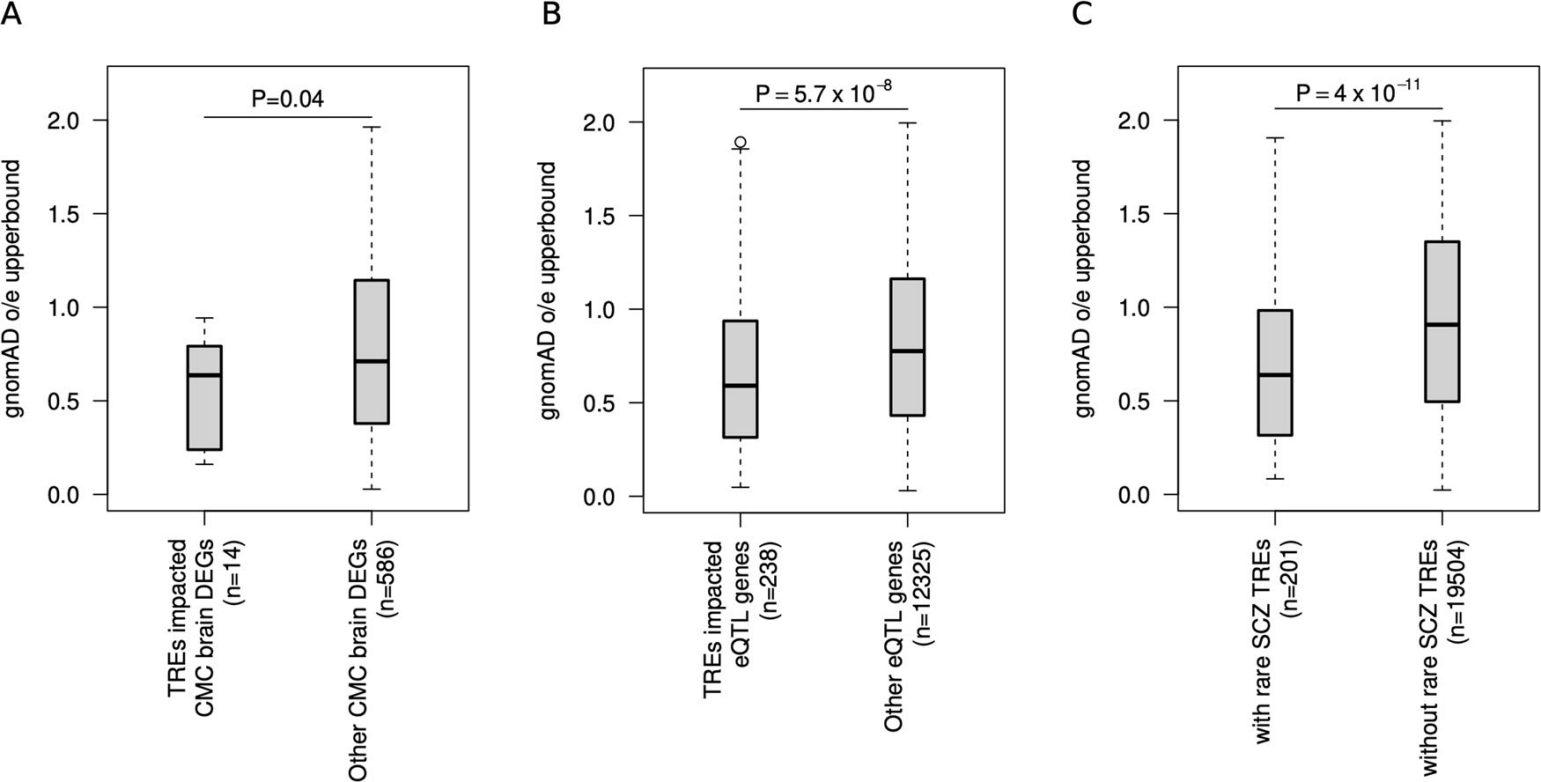
Constraint scores for genes with and without rare TREs. Constraint scores (extracted from gnomAD’s observed/expected (o/e) upper bound LOEUF values) of genes with rare TREs are compared against those of the other genes without rare TREs in schizophrenia. **A** Only genes differentially expressed between schizophrenia and controls as determined by the Common Mind Consortium are compared, **B** only genes with eQTL are compared and (**C**) comparison is done for all protein coding genes. Box plots show Q1-1.5×IQR, Q1, median, Q3 and Q3 + 1.5×IQR. P-values reported were calculated from one-sided Wilcoxon rank-sum test assuming lower gnomAD o/e upperbound in genes with rare TREs. In all three categories assessed, the genes with rare TREs found in schizophrenia were more constrained (i.e., had on average lower LOEUF values) than the genes without rare TREs found in schizophrenia.

To further explore the potential mechanisms by which TREs may regulate the underlying genes, we compared the level of mutational constraints (determined by the observed over expected number of loss of function variants in gnomAD [48]) within CMC DEGs, within brain eQTL genes and in all genes with and without rare TREs found in schizophrenia cases. We found that rare TREs impacting CMC brain DEGs were significantly more constrained than other CMC brain DEGs (Fig. 4a) and rare TREs impacting genes with eQTLs are significantly more constrained than other CMC brain DEGs (Fig. 4b). We also found that the genes with rare TREs found in schizophrenia cases were significantly more constrained than genes without rare TREs found in schizophrenia cases (Fig. 4c).

Our study is underpowered to detect individual loci of rare TREs associated with schizophrenia at genome-wide significance (Supplementary Fig. 8). We compared the individual rare TRE loci from our data to the 39 top-ranking rare TRE loci identified by Mojarad et al. [18]. Nine of the 39 loci were found in the schizophrenia cases from this study (Supplementary Table 11). To explore whether pleiotropic TRE loci may exist across ASD and schizophrenia, we compared the individual rare TRE loci from our data to the top 57 rare TRE loci identified by Trost et al that were in the top two gene sets enriched in ASD and had a higher frequency in individuals with ASD than siblings without ASD [16]. 14 of the 57 ASD loci were found in the schizophrenia cases from this study (Supplementary Table 11).

### Confirmation of EHdn detected tandem repeats

**Fig. 5:**
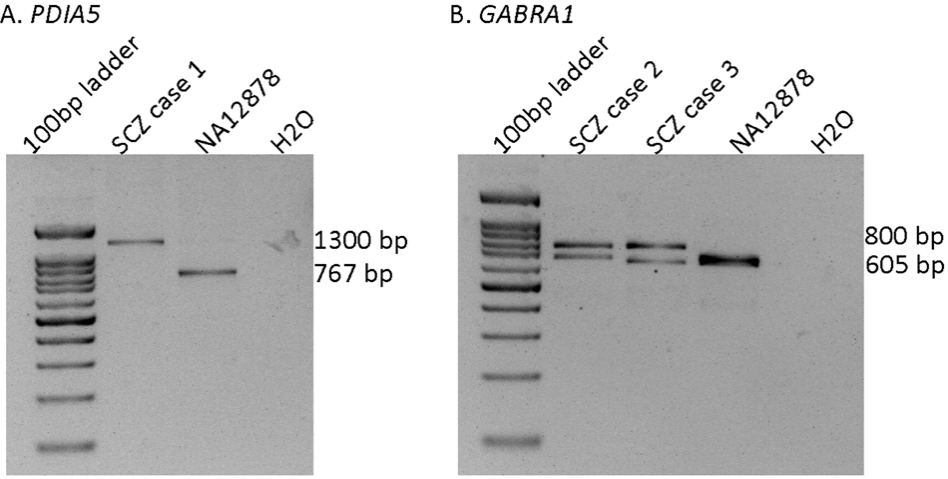
Confirmation of EHdn detected tandem repeats. **A**, **B**, Images of the gel electrophoresis showing bands that correspond to the expanded alleles in schizophrenia (SCZ) cases and the unexpanded allele in the reference sample NA12878. A 100 bp ladder is shown for size reference.

The detected tandem repeats from EHdn were validated using multiple complementary methods by Trost et al, which showed that the validation rate of detected tandem repeats was 77% compared to long-read sequencing [16]. In this study, we used an identical pipeline as Trost et al. [16], so we expected the same rate of validation in our calls. Furthermore, we selected two TRE regions for validation based on (1) high odds ratio from burden testing, (2) located close to genes of interest (i.e. loss of function intolerant genes and/or known risk genes for schizophrenia), and (3) absence of complex repetitive sequences nearby. We attempted to confirm TREs in the chosen regions by Integrative Genomics Viewer (IGV) visualization of WGS reads and gel electrophoresis size separation of PCR products of the regions of interest. All TREs detected were confirmed (Fig. 5 and Supplementary Fig. 10).

### Replication

We sought replications for significant associations detected in our Swedish data in an independent dataset from Canada that consisted of 252 schizophrenia cases of European ancestry and 222 ancestry-matched controls [18]. We excluded post-synapse genes from final testing because the number of rare TREs observed in this gene-set in the replication samples was too small (<5). For each of the remaining significant associations, we conducted an association analysis in the replication samples and then a meta-analysis of the Swedish and independent Canadian samples for the total sample size of 1,436 schizophrenia cases and 1,156 controls. As shown in Supplementary Table 12, there is a high concordance between discovery and replication samples and all significant associations detected in our discovery samples have been replicated.

## Discussion

We have carried out one of the first genome-wide investigations of rare TREs in schizophrenia. We found a significant excess of rare TREs in schizophrenia in genic regions, in genes with brain eQTLs, in brain-expressed genes that are differentially expressed between schizophrenia cases and controls, and in genes involved in postsynaptic processes. These results have been replicated in independent samples [18]. Our results suggest that rare TREs collectively play a role in schizophrenia etiology.

We estimated ~7% excess of rare TREs in schizophrenia compared to controls, which may approximate their collective contribution to schizophrenia risk. This is modest compared to the estimated contribution of common variation to schizophrenia risk [5], but is comparable in magnitude to the previously estimated contribution of tandem repeat expansions to disease risk in schizophrenia and ASD [16, 17]. Mojarad et al estimated that rare exonic TREs contribute to ~4% of the risk in schizophrenia based on excess of variants detected [18]. However, we note that we employed a more stringent rare variant frequency filter (0.1%) than that used in Mojarad et al (0.5%), and, therefore, the higher excess observed in our study is as expected [18]. Trost et al and Mitra et al estimated that rare TREs contribute to ~4% of ASD cases based on excess of variants detected [16, 17]. It is noteworthy however that the risk estimation from rare TREs in ASD was based on comparison of sample proportions between affected probands and unaffected siblings within families, while our risk estimation in schizophrenia was based on comparison of sample proportions between unrelated individuals. The risk estimation in ASD may be more conservative as some unaffected siblings within ASD families may have inherited shorter, yet expanded, tandem repeats [16]. Apart from the comparable collective contributions from rare TREs to ASD and schizophrenia, we did not identify with statistical significance TRE gene-pathways shared between ASD and schizophrenia, though we were likely underpowered to detect small enrichment given the rarity of the variants.

Our finding that rare TREs are enriched in schizophrenia in brain expressed genes with eQTLs or with different expression levels between schizophrenia cases and controls is not surprising. Tandem repeats can directly affect coding sequences or play an important role in the regulation of gene expression via a variety of mechanisms [7, 12]. The functional profiles of the implicated genes were found to involve synaptic functions and signaling which is consistent with a prior report of tandem repeats in schizophrenia [18]. Our finding that genes with TREs tend to be more constrained is consistent with the literature [18] and suggests that TREs may affect the underlying genes in the same manner as loss of function variants. Our finding that rare TREs are enriched in schizophrenia in genes involved in postsynapse process, a gene set indicated by the largest schizophrenia GWAS [5], suggests that studies of tandem repeats may pinpoint shared underlying biology that is dysregulated across the spectrum of variant type and allele frequency. As our present study is correlative, a future direction would be to test the role of schizophrenia-associated TREs in patient-derived stem cells and model organisms in order to understand their precise functional effects on gene expression.

Although we observed a mildly elevated burden of rare TREs in schizophrenia in non-coding conserved bases, we did not identify significant enrichment of rare TREs within various categories of active regulatory elements in human brain. We note that there may be a disproportionately low representation of regions containing tandem repeats in currently available functional genomic annotations. For example, the use of exclusive regions of “blacklists” have been employed by the ENCODE project to remove signal artifact regions in next-generation sequencing experiments [34, 49]. These blacklisted regions may be due to repetitive elements or other anomalies where genome assembly has been difficult resulting in problematic read alignment and erroneous signals. Such blacklist filtering is a widely-used quality control step for functional genomics assays.

We acknowledge several constraints in variant detection owing to limitations in existing algorithms. First, EHdn detects tandem repeats only with motifs of 2-20 bp and total length larger than >150 bp. We were unable to evaluate tandem repeats with motif size or total length beyond the detection ranges. Second, as demonstrated by Trost et al. [16], EHdn likely underestimated the number of repeat units for larger tandem repeats when the total length of a tandem repeat is greater than the sequencing insert size (approximately 350 bp for the Swedish schizophrenia cohort). Third, very small expansions, such as expansions with one or two additional repeat units, are missed. A few such small TREs, such as those found in spinocerebellar ataxia types 1, 2, and 6, are known to be disease causing [7, 50]. Fourth, our method does not resolve zygosity or orientation of the tandem repeats (only an aggregated length was estimated).

With the current sample size, we lack the power to detect individual tandem repeat loci that are associated with schizophrenia risk at genome-wide significance. Prior studies in ASD that had sample size larger than ours but in similar magnitude (~2000 cases) also failed to implicate individual loci of tandem repeats with ASD risk [16, 17]. Much larger cohorts (on the order of N_case/control_ > 10,000), combined with future improvements in variant detection methods, will likely pinpoint specific tandem repeat loci [19]. Substantial collaborative efforts will be critical in the pursuit of larger sample sizes. Our data are meant to be included through such collaborative efforts in the future in meta-analyzing whole genome sequence datasets in schizophrenia and other psychiatric disorders.

## Supplementary information

Supplementary Methods and Figures

Supplementary Tables
