# Supplementary material for: Rare tandem repeat expansions associate with genes involved in synaptic and neuronal signaling functions in schizophrenia
Source: Mol Psychiatry. 2022 Nov 16;28(1):475–82. doi: 10.1038/s41380-022-01857-4 (PMC9812781; doi:10.1038/s41380-022-01857-4)
Supplement: Supplementary file 1 — Supplementary Methods and Figures [file 41380_2022_1857_MOESM1_ESM.pdf]

Supplementary Methods and Figures

Rare tandem repeat expansions associate with genes involved in synaptic and neuronal signaling functions in schizophrenia

Wen [#], Trost [#], Engchuan [#] et al.

Table of Contents

Supplementary Methods **2**

Genome-wide detection of tandem repeats 2

Quality control 2

Detection of tandem repeat expansions 3

Genome annotations in burden testing 3

Burden of rare tandem repeat expansions 5

Replication 6

Gene constraint analysis 7

Gene network analysis 7

Power calculation and correction for multiple comparisons 7

Data Availability 8

Code Availability 8

Supplementary Figures **9**

Supplementary Figure 1. Sample-level quality control 9

Supplementary Figure 2. The number of unique motifs in each repeat-containing region. 10

Supplementary Figure 3. The distribution of motifs 11

Supplementary Figure 4. The distribution of motif size 12

Supplementary Figure 5. Correlation analysis between tandem repeats and GC content/sequence conservation 13

Supplementary Figure 6. Novel tandem repeats 14

Supplementary Figure 7. Distribution of the count of rare tandem repeat expansions 15

Supplementary Figure 8: Power calculation 16

Supplementary Figure 9 Principal Component Analysis 17

Supplementary Figure 10 IGV read pile-up in regions of interests 18

Supplementary Methods

Genome-wide detection of tandem repeats

We used ExpansionHunter Denovo (EHdn)[1] (https://github.com/Illumina/ExpansionHunterDenovo), an efficient catalog-free method for genome-wide tandem repeat detection from short-read whole-genome sequence data. EHdn scans the read alignment file (i.e., BAM/CRAM) of a given sample to search for anchored in-repeat reads (IRRs), defined as paired reads where one mate is completely contained inside the repeat and the other "anchored" mate is confidently aligned to the non-repetitive genomic sequence adjacent to the repeat. The genomic coordinates where the anchored reads align provide estimates of the locations of the tandem repeats, whereas the number of anchored IRRs provide estimates of the sizes of the tandem repeats. EHdn v0.7.0 was run with default parameters on each sample and one sample at a time (1,159 schizophrenia cases, 936 controls, and 2,504 1000GP samples), producing per-sample output files containing information about anchored IRRs for each repeat motif (i.e., repeat units, regions containing anchored IRRs in close proximity to each other, and counts of anchored IRRs). The input read alignment BAM files for the Swedish schizophrenia case and control were aligned to the GRCh37 human reference genome using bwa mem, whereas the input BAM files for the 1000GP samples were aligned to GRCh38 using bwa mem. We lifted over the EHdn output files for the Swedish schizophrenia cases and controls from hg19 to hg38 to be consistent with the 1000GP samples. All genomic coordinates are thus given in NCBI Build 38/UCSC hg38.

We then combined the per-sample output using the combine_counts.py script provided with EHdn and generated the final set of regions using the compare_anchored_irrs.py script with the parameter minCount = 2. During this process, nearby anchored IRR regions were merged across multiple samples, and the associated anchored IRR counts were normalized by the average sequencing depth of the sample (hereafter referred to as normalized anchored IRR counts).

Quality control

All Swedish schizophrenia cases and controls included in this work for tandem repeat detection have passed rigorous quality control procedures carried out in our previous study[2]. All subjects were unrelated (pairwise relatedness $\hat{\pi} < 0.2$), had no presence of sex mismatch or sex chromosome abnormality, and had SNV/indel missing rate per sample < 0.01 and heterozygosity rate < 0.1. Principal component analysis was done previously following established pipelines using eligible bi-allelic autosomal SNPs which confirmed the relative homogeneity of the sample. For the current analysis, we further evaluated the distribution of EHdn-detected tandem repeat counts using histograms and QQ plots and found that a normal distribution was best approximated by removing outlier samples with a count >3 standard deviations from the mean (Supplementary Figure 1). All 1000GP samples have passed quality controls conducted in Byrska-Bishop et al[3].

To determine whether the number of tandem repeats detected in a given sample was affected by systematic biases in the sequencing data, we applied a principal component analysis to the normalized anchored IRR counts in schizophrenia cases and controls and plotted the first two principal components.

Detection of tandem repeat expansions

Analysis was done using R (3.6.0 & 4.0.3). Following [4], we applied the density-based spatial clustering of applications with noise (DBSCAN) algorithm to identify tandem repeat expansions (TREs) whose lengths were outliers compared with other members of the cohort. DBSCAN is a non-parametric clustering algorithm and thus no assumption about the size distribution is required. DBSCAN defines a cluster based on the minimum number of data points (minPts) reachable to each other by a maximum distance (ε). Tandem repeats not reachable by the clusters and larger than any cluster members are classified as outliers or tandem repeat expansions. The DBSCAN parameter minPts was set to be −log2 (n) ≈ 15 (where n is the number of samples and is 4,592), and ε was set to be 2 × Mo(X_i) (where Mo is the mode and X_i is a vector of tandem repeat sizes for repeat i). EHdn only detects tandem repeats larger than the sequence read length, so as suggested by[4], we simulated the sizes of repeats not meeting this criterion using a normal distribution with a mean of 1, a standard deviation of 0.25, and a maximum of 2.

We defined rare TREs as TREs that were found in less than 0.1% of the 1000GP population controls.

Genome annotations in burden testing

We annotated tandem repeats with gene definition and gene part annotations (CDS, intron, splice site, 5'UTR, 3'UTR, promoter ± 2000) from GENCODE v36.

For conserved DNA sequences, we used the phyloP annotations identified by the Zoonomia project, bases constrained across the evolution of 240 mammals (with phyloP scores exceeding the FDR 0.05 threshold ≥ 2.270, about 3.53% of the genome)[5].

For brain epigenomics annotations, we relied on empirically generated annotations that have been shown to be important to gene regulation in the brain. Epigenomic data are restricted to the autosomes and were originally developed in hg19. We lifted all positions to hg38 for the present study.

- ATAC-seq_AdultBrain: Open chromatin regions obtained from ATAC-seq on adult prefrontal cortex brain samples as reported in [6]. ATAC-seq was performed on adult prefrontal cortex brain samples from 135 individuals with schizophrenia and 137 controls.
- CTCF from neural cells: CTCF from neural cells was downloaded from GSE127577 ENCODE [7].
- Sub-TAD boundaries in AdultBrain: Boundaries of topologically associating domains (TADs) obtained from "easy-HiC" on postmortem adult temporal cortex samples as described in [8], and TAD boundaries were defined in 40 kb bins. To generate sub-TAD boundaries, we have applied an optimized nested TAD caller termed OnTAD [9] on TADs from [8] to identify hierarchical TADs. The OnTAD output included the level of each TAD in the hierarchy with small value denoting outer TADs and large value denoting inner TADs/sub-TADs. We then ran run burden testing for level-1 TAD and level-2 TAD (i.e. sub-TAD) boundaries separately.

- H3K27ac_Neurons: As described in Girdhar et al[10], chromatin immunoprecipitation and sequencing was performed with anti-H3K4me3 and anti-H3K27ac antibodies, and separately for neuronal and non-neuronal nuclei extracted from two frontal lobe areas, the dorsolateral prefrontal cortex and the anterior cingulate cortex. We used the neuronal differential cell specific H3K27ac peaks for the present study.
- H3K4me3_Neurons: Neuronal differential cell specific H3K4me3 peaks as described in [10].
- FIREs_Neurons: As described in Hu et al[11], Hi-C data was generated from Neurons (NeuN+ cells) and glia (NeuN− cells) sorted from four dorsolateral prefrontal cortex samples, from which FIREs and super-FIREs were defined at 40 kb resolution. For the present study, we extracted Supplementary Data 1 of Hu et al[11], NeuN+.
- super-FIREs_Neurons: Supplementary Data 2 of Hu et al[11], NeuN+.
- Enhancer-promoter interactions_Glu/GABA-Neurons: Hu et al[11] used H3K27ac peaks defined in purified Glu and MGE-derived GABA (referred to as GABA) neurons to deconvolute NeuN+ chromatin interactions into two major neuronal subtypes. For the present study, we extracted Supplementary Data 4 of Hu et al[11].
- Anchors based on promoter-based interactions NeuN+: [11] All anchors defined by promoter-based interactions in NeuN+, obtained from Hu et al [11].

For schizophrenia (SCZ) risk genes, we assessed gene sets previously implicated in schizophrenia from GWAS, copy number variation, exome sequencing, and gene expression studies including: CMC_differentially_expressed_genes: The CommonMind Consortium (CMC) sequenced RNA from dorsolateral prefrontal cortex of schizophrenia cases (N = 258) and control subjects (N = 279), from which we selected genes implicated to have differential expression in human brain between cases and controls based on qvalue < 0.05[12].

- LOF_intolerant_genes: Loss-of-function intolerant genes from [13].
- CHD8-targeted promoters_Cotney et al: we used genes from [14].
- Targets of FMRP_Darnell et al: we used the 842 mouse genes from Supplementary Table 2A of [15], including all genes with FDR < 0.01.
- SCZgenes_rarevar_SCHEMA_p001: The Schizophrenia Exome Sequencing Meta-Analysis (SCHEMA) consortium identified risk genes with damaging ultra-rare mutations associated with schizophrenia, from which we selected genes based on P value < 0.001[16]. When more stringent thresholds (genome-wide significance or FDR<0.05) were used, we did not observe overlapping tandem repeat expansions.
- Genes_in_SCZ.CNVs: Genes in any schizophrenia-associated CNVs identified by The Psychiatric Genomic Consortium (PGC)[17].
- SCZgenes_commonvar_PGC3: 125 prioritized schizophrenia risk genes identified by the PGC wave 3 genome-wide association study (GWAS). The list of genes was lifted from Extended Data Table 1 of [18].
- Synapse - GO:0045202: SynGO ontology associated with schizophrenia risk identified by the PGC wave 3 GWAS [18].
- Postsynaptic_membrane - GO:0045211: SynGO ontology associated with schizophrenia risk identified by the PGC wave 3 GWAS [18].

- Postsynaptic_density_membrane - GO:0098839: SynGO ontology associated with schizophrenia risk identified by the PGC wave 3 GWAS[18].
- Postsynapse - SYNGO:postsynprocess: SynGO ontology associated with schizophrenia risk identified by the PGC wave 3 GWAS[18].
- Synaptic_signaling - GO:0099536: SynGO ontology associated with schizophrenia risk identified by the PGC wave 3 GWAS[18].
- Synapse_organization - GO:0050808: SynGO ontology associated with schizophrenia risk identified by the PGC wave 3 GWAS[18].
- For risk genes associated with neurodevelopmental disorders including autism spectrum disorders (ASD), developmental delay disorders (DDD), and intellectual disability (ID), we assessed gene sets previously implicated in schizophrenia from copy number variation and exome sequencing studies, as well as studies of tandem repeat expansions.
- ASDgenes_Satterstrom et al: ASD risk genes identified by exome sequencing study Satterstrom et al[19]
- DDDgenes_Kaplanis et al: DDD risk genes identified by exome sequencing study Kaplanis, J. et al[20]
- IDgenes_Kochinke et al: ID risk genes identified by exome sequencing study Kochinke et al[21]
- Genes_in_ASD.DDD.ID_CNVs: Genes in any pathogenic CNVs associated with the risk of ASD, DDD, or ID from [22-24]
- Nervous system development - GO:0007399: This geneset is GO:0007399 nervous system development, which was enriched for ASD-linked tandem repeat expansions[4]
- MPO: Cardiovascular or muscle abnormality: This is the union of two MPO-based gene-sets: MP:0005385 cardiovascular system phenotype, and MP:0005369 muscle phenotype, which was enriched for ASD-linked tandem repeat expansions[4]
- ASDgenes_SFARI: Known ASD-risk genes from the Simons Foundation Autism Research Initiative (SFARI) gene database, in which Trost et al found that the burden of rare tandem repeat expansions was significantly more pronounced in ASD[4]
- Genes with eQTL from PsychENCODE Integrative Analysis: eQTLs with gene expression > 1 FPKM in at least 20% of the samples from PsychENCODE Integrative Analysis (downloaded from http://resource.psychencode.org/). Finally, we in total had 16,503 genes with eQTLs for burden analysis in our study [25]

Burden of rare tandem repeat expansions

Analysis was done using R (v4.0.3) using a logistic regression framework established in prior studies[4]. Logistic regression models are clarified below. To identify potential variables that can confound burden tests, we evaluated 9 PCs calculated from normalized IRR counts, that is PC2 through PC10. First, we estimated the proportion of variance explained by each of the PCs under consideration from the principal component analysis of the normalized anchored in-repeat reads. We found that 7.1% of variance was explained by PC2, 3.4% by PC3, and around 1% for PC4 to PC10. Second, we fitted logistic regression models with case/control status as the dependent variable and each of the PCs under consideration as the independent variable. Of the 9 PCs considered, we found that PC2, PC3 and PC8

were significantly associated with case/control status. Third, we performed a sensitivity analysis to evaluate how burden testing results in intergenic regions can be affected based on changes in the covariates in logistic regression models. We used burden in intergenic regions for benchmarking in the sensitivity analysis because we expected null result (i.e. its odds ratio close to one) based on a previous report that there was no case control difference in burden of rare TREs in intergenic regions in autism spectrum disorders (odds ratio = 1.010, p value = 0.905 [4]). As summarized in the Supplementary Table 13, we found that (a) including PC2 as a covariate brought the adjusted odds ratio for intergenic burden close to one (comparing model 2 vs 1); (b) including additional PCs resulted in minor difference in burden estimates but no change in P values compared to that based on including PC2 alone (models 3 or 4 vs 2). This analysis suggests that although our original model with PC2 was adequate, the most defensive method would be to include all three significant PCs (i.e. PC2,PC3, and PC8) in logistic regression models. Finally and importantly, when we performed hypothesis testing in specific regions (i.e. genic regions, gene sets, epigenomic annotations), we included intergenic burden as a covariate to further control for any confounding effect that was not captured by the PCs. Therefore, for genome-wide burden analysis of rare TREs and intergenic rare TREs, we fit the following logistic regression model: y ~ covariates + global, where y is the outcome phenotype variable (schizophrenia=1, control=0), covariates include sex and the PC2, 3 and 8, and global is the genome-wide total number of rare TREs. For all burden tests in target regions (including genic TREs, conserved sequences, different parts of genes, gene sets of interest, brain epigenetic annotations), we fit the following logistic regression model for each target region: y ~ covariates + intergenic_burden+ target_region, where covariates are the same as before, intergenic_burden is the count per sample of intergenic rare TREs, and target_region is the count per sample of rare TREs specific to the target region annotation. We included intergenic to increase the specificity of the tests in target regions. For all tests, we carried out one-sided statistical tests assuming an increased burden of rare TREs in schizophrenia. Logistic regression models were implemented by the glm function in R (v4.0.3). Odds ratios were computed to measure the increase in the likelihood of having disease per unit increase in the burden of rare TREs.

We estimated the proportion of samples carrying rare TREs using the residuals of rare TRE counts after controlling for confounding factors. First, we fit a regression model of covariates (sex and PC2, PC3, and PC8 of the normalized anchored IRR counts) to rare TRE counts assuming Gaussian distribution, and then extracted the residual values defined as the residuals of rare TRE counts after controlling for confounding factors. Next, we created a binary indicator variable based on the residuals, which was assigned a value of 1 if a residual value was greater than or equal to 1 and assigned a value of 0 if otherwise. Finally, we estimated sample proportions in cases and controls separately by calculating the sum of the binary indicator variable divided by the total number of samples. The Wilcoxon ranked sum test was used to compare sample proportions.

Replication

We obtained replication association results from an independent dataset from Canada that included 252 unrelated adult cases with schizophrenia of European ancestry and 222 ancestry-matched individuals with no major neuropsychiatric disorders[26, 27]. Genome-sequencing of all replication samples were performed at The Centre for Applied Genomics (TCAG, Toronto, Canada) using library prep kits and

sequencing platform that are the same as those used for our Swedish schizophrenia cases and controls (TruSeq DNA library prep kits, Illumina HiSeq X platform with 2 × 150 bp paired-end reads). Sequence data were processed for read alignment. TRE identification used the same pipeline using ExpansionHunter Denovo (Ehdn) and used 2504 individuals from 1000 Genomes Project to estimate population frequency of TREs[26]. From study [26], a total of 1221 rare TREs (<0.5% population frequency) were identified in these schizophrenia cases and controls. We annotated those rare TREs using CMC DEGs, genome-wide genes, and genes with eQTLs from PsychENCODE Integrative Analysis, using methods identical to those for the Swedish sample. Then we performed a burden analysis using a logistic regression model by regressing the case-control status versus the number of rare TREs of interest and using sex and total number of rare TREs per subject as covariates, as previously done in [26]. We only included rare TREs on the autosomal chromosomes in the burden analysis and one-sided Wald Test P from the logistic regression model was reported, assuming an increased burden in schizophrenia cases than controls. We then used METAL [28] to perform a fixed effect meta-analysis using the inverse variance-based method to meta-analyze the results between the original and the replication studies.

Gene constraint analysis

We obtained the upper bound 90% confidence intervals of constraint score (ratio of the observed / expected (oe) number of loss-of-function variants) in genes from gnomAD version 2.1.1[29]. We applied Wilcoxon signed rank test (one-sided) to test whether the genes (all genes, CMC DEGs and genes with eQTLs) with rare TREs have lower constraint scores than the other genes.

Gene network analysis

For the gene network analysis, the CMC differentially expressed genes and known schizophrenia genes (from Marshall et al 2017[17], SCHEMA FDR < 0.05[12], and PGC3 GWAS[18]) were used as baits to query the Genemania[30] database using the Cytoscape plugin[31] (database version: homo sapiens 2021-04-29) limiting the interaction networks to pathway and physical interactions with 50 top genes and GO biological process-based weighting, and visualized in Cytoscape v3.8.1[32]. Genes with no interaction partners were removed from the results. Genes with strong connections and involved in functionally related categories were manually edited and adjusted for visualization purpose.

Power calculation and correction for multiple comparisons

We used the R/gap package (v1.2.1, https://github.com/jinghuazhao/R) to estimate statistical power for association analyses. We assumed an additive model, lifetime risk of schizophrenia of 1%, and two type I error levels: (1) $5x10^{-8}$ as an established genome-wide significance threshold for single variant association, (2) $1x10^{-5}$ as in [33]. We computed the minimal detectable genotypic risk ratio to achieve 20%, 80% power over a range of frequency of risk alleles in the population. For the burden test, the X-axis of the power plot (Supplementary Figure 8) represents the aggregated frequency of a set of variants aggregated for a target region of interest. To correct for multiple testing, we applied the Benjamini and Hochberg false discovery rate (BH-FDR) method across all 38 burden tests performed (Supplementary

Table 4-6). Following [4], we used a threshold of 0.10 on the FDR adjusted P values (a.k.a. q values) to evaluate statistical significance.

Data Availability

Tandem repeat regions identified from this study as well as all other summary statistics and supporting data are available in Supplementary Information. The 1000G genome-sequencing data are publicly available via Amazon Web Services (s3://1000genomes/1000G_2504_high_coverage/data). Due to recent changes in Swedish and European Union regulations regarding genetic data, we are unable to deposit individual-level data for the Swedish individuals into controlled-access repositories like dbGaP. Collaborative analyses are possible and can be pursued by contacting the authors.

Code Availability

Analysis software used in this study include the following: R (v4.0.0, https://www.r-project.org); R/gap package (https://github.com/jinghuazhao/R); Python 3.7.2; ExpansionHunter Denovo: ExpansionHunterDenovo-v0.7.0 (https://github.com/Illumina/ExpansionHunterDenovo); BEDTools 2.27.1 (https://bedtools.readthedocs.io/en/latest/); Genemania (https://genemania.org/); OnTAD v1.4 (https://github.com/anlin00007/OnTAD).

Supplementary Figures

Supplementary Figure 1. Sample-level quality control

We examined the distributions of total tandem repeats counts across schizophrenia cases and controls after excluding samples that lie outside of 1x, 2x, 3x, or 4x standard deviations (SD) of the mean count. Histograms and QQ plots showed that a normal distribution of tandem repeat counts was best approximated by pruning samples having a count >3x SD from the mean.

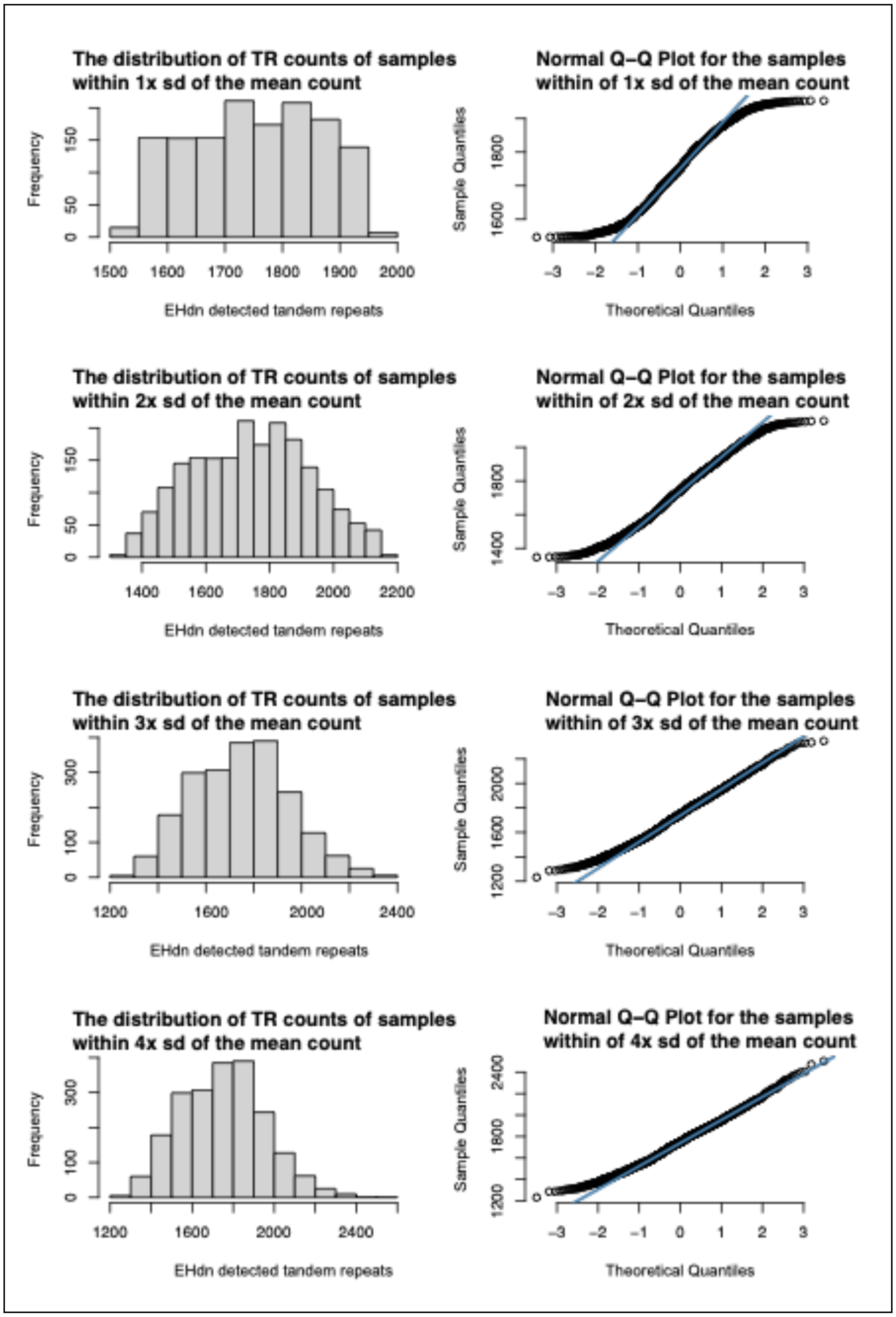

Supplementary Figure 2. The number of unique motifs in each repeat-containing region.

The number of unique motifs (y axis) in each repeat-containing region (x axis) is shown for all autosomal chromosomes and chromosomes X and Y. The average number of motifs per region was 1.26; 14,848 regions (88.8%) had one motif and 1,875 (11.2%) had ≥ 2. As many as 66 different motifs were observed in a single region on chromosome 2 (chr2:32916196-32916582), which overlapped the peak region identified in a previous report (Trost et al[4], chr2:3291589-32916586).

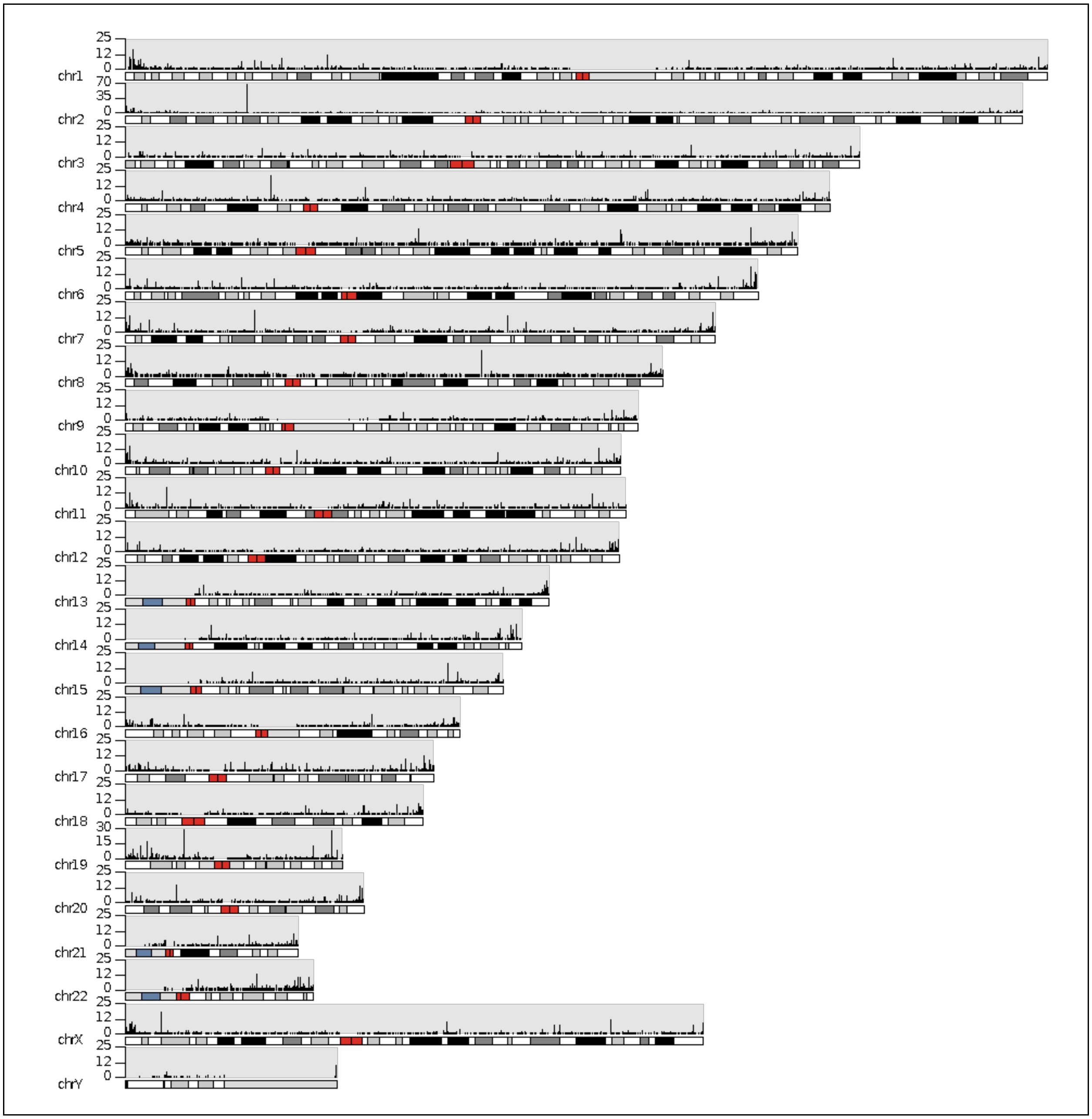

Supplementary Figure 3. The distribution of motifs

This figure shows the 20 most common repeat motifs. The motifs were AC- and AG-rich, with AC the most common motif (17.5%).

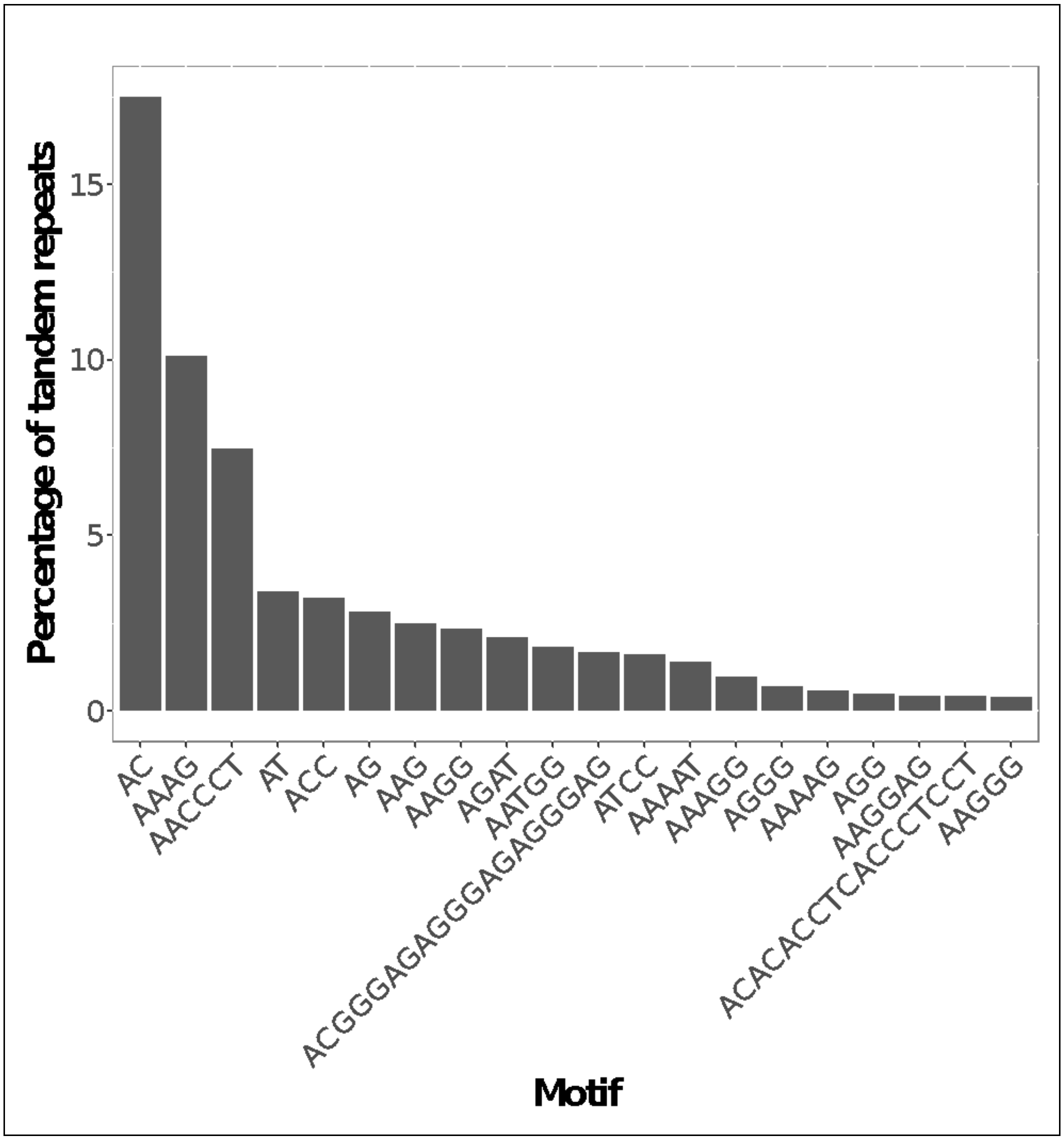

Supplementary Figure 4. The distribution of motif size

The most common motif size was 2 bp, found in 23.7% of the repeat tracts. The majority (66.01%) of repeat tracts had motifs between 2 and 6 bp, with even-numbered motif sizes observed more frequently than odd-numbered sizes.

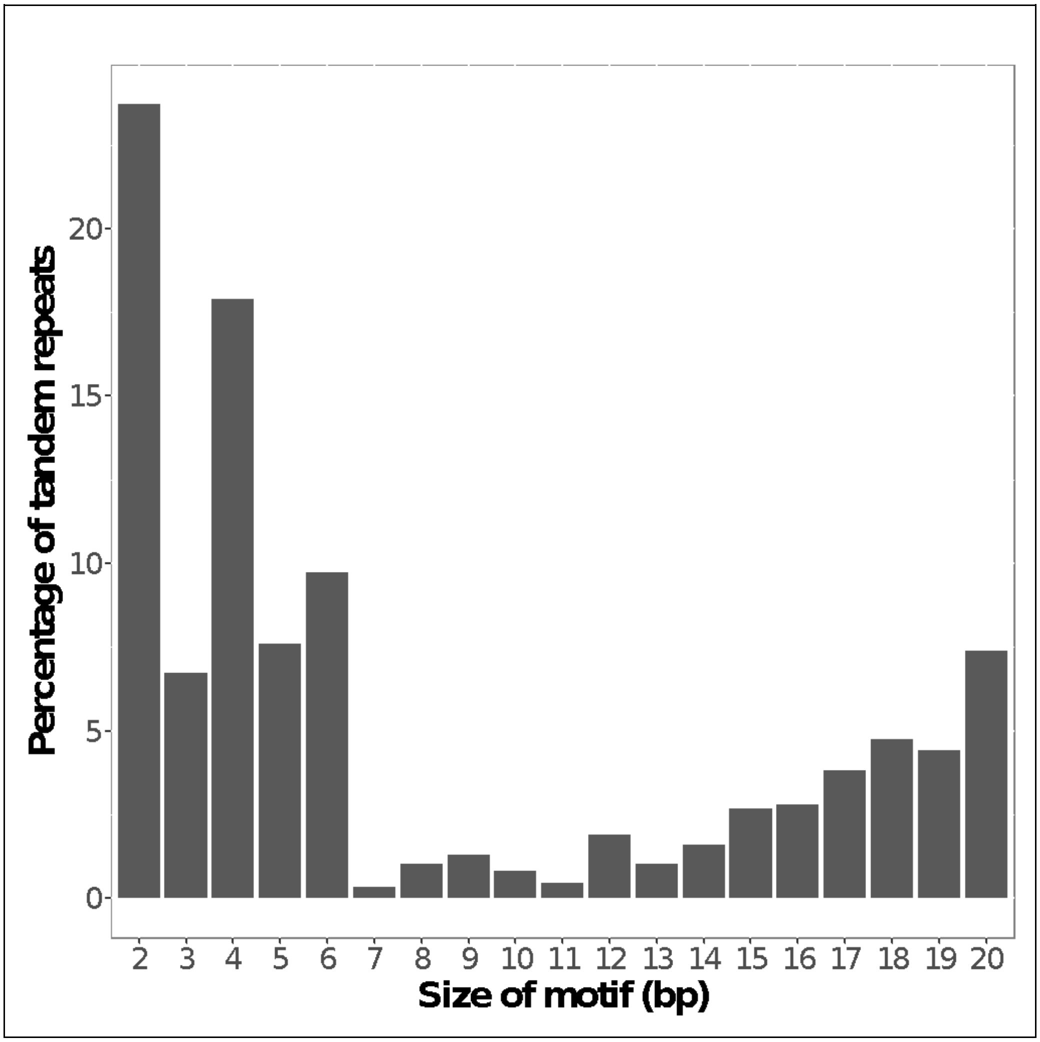

Supplementary Figure 5. Correlation analysis between tandem repeats and GC content/sequence conservation

Following Trost et al[4], we binned the genome into 1-kb windows and tested the correlation of tandem repeats and genomic features including GC content and two measures of sequence conservation as defined by PhyloP[5] and PhastCon[34]. The correlation analysis was based on a logistic regression framework by regressing a genomic feature on the number of tandem repeats found per window. Y axis denotes odds ratio derived from logistic regression. We performed the analysis for tandem repeats detected in this study (red bar) and for known simple sequence repeats (blue bar).

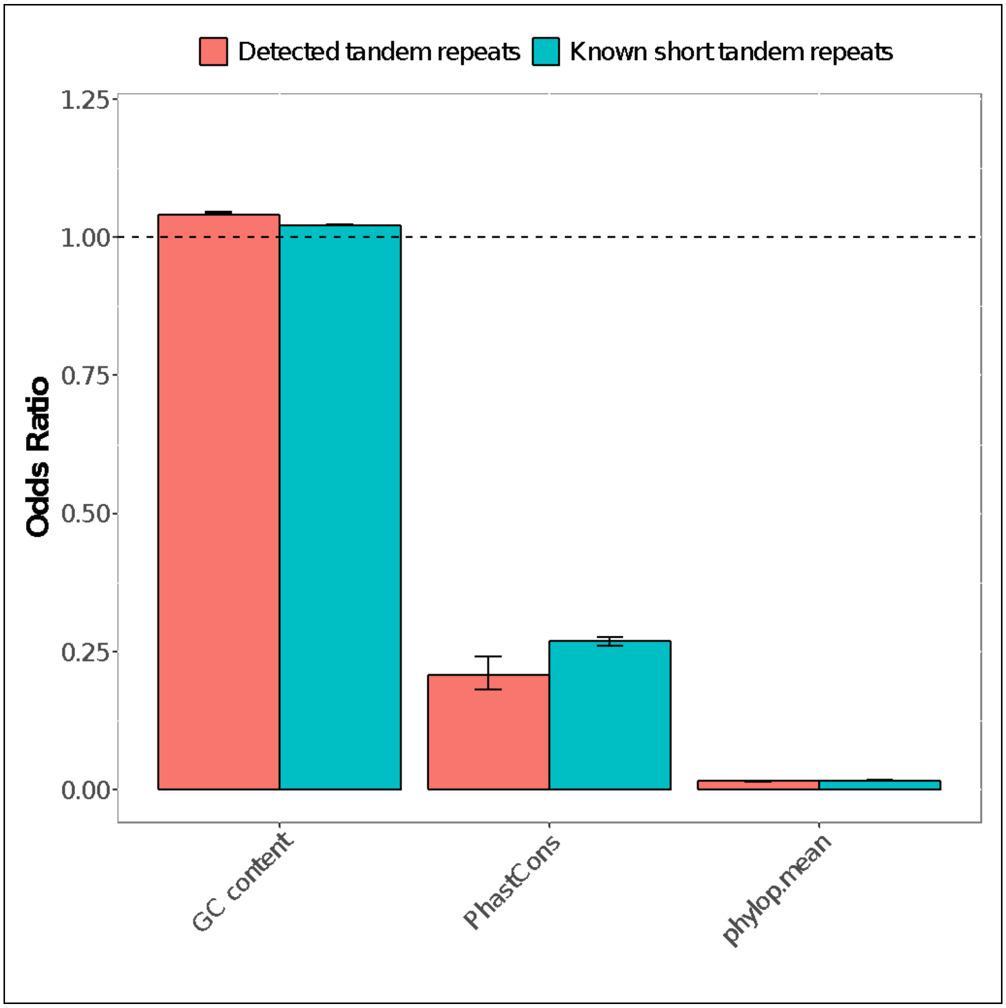

Supplementary Figure 6. Novel tandem repeats

We compared our data to the known simple sequence repeat regions in the human reference genome and to tandem repeat loci reported In Trost et al[4]. Of the 16,723 tandem-repeat-containing regions reported in this study, 5428 overlapped (1 base pair overlap) regions reported in both Trost et al[4] and known simple sequence repeats, 3427 overlapped (1 base pair overlap) regions reported in Trost et al only but not in known simple sequence repeats, 4421 overlapped (1 base pair overlap) regions reported in known simple sequence repeats only but not in Trost et al, and 3447 (20.6%) regions repeated in this study have not been previously reported (i.e. they are novel).

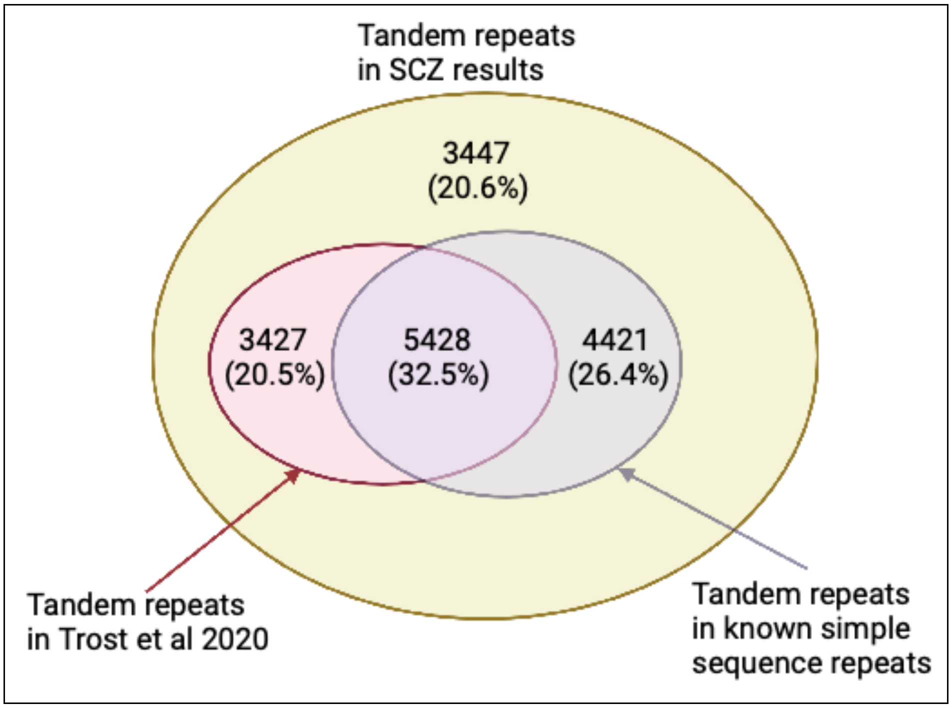

Supplementary Figure 7. Distribution of the count of rare tandem repeat expansions

We examined the distribution of the count of rare tandem repeat expansions per sample using a stratified histogram where the red color denotes schizophrenia cases and the blue color denotes the controls. We did not observe outliers based on the count of rare tandem repeat expansions per sample.

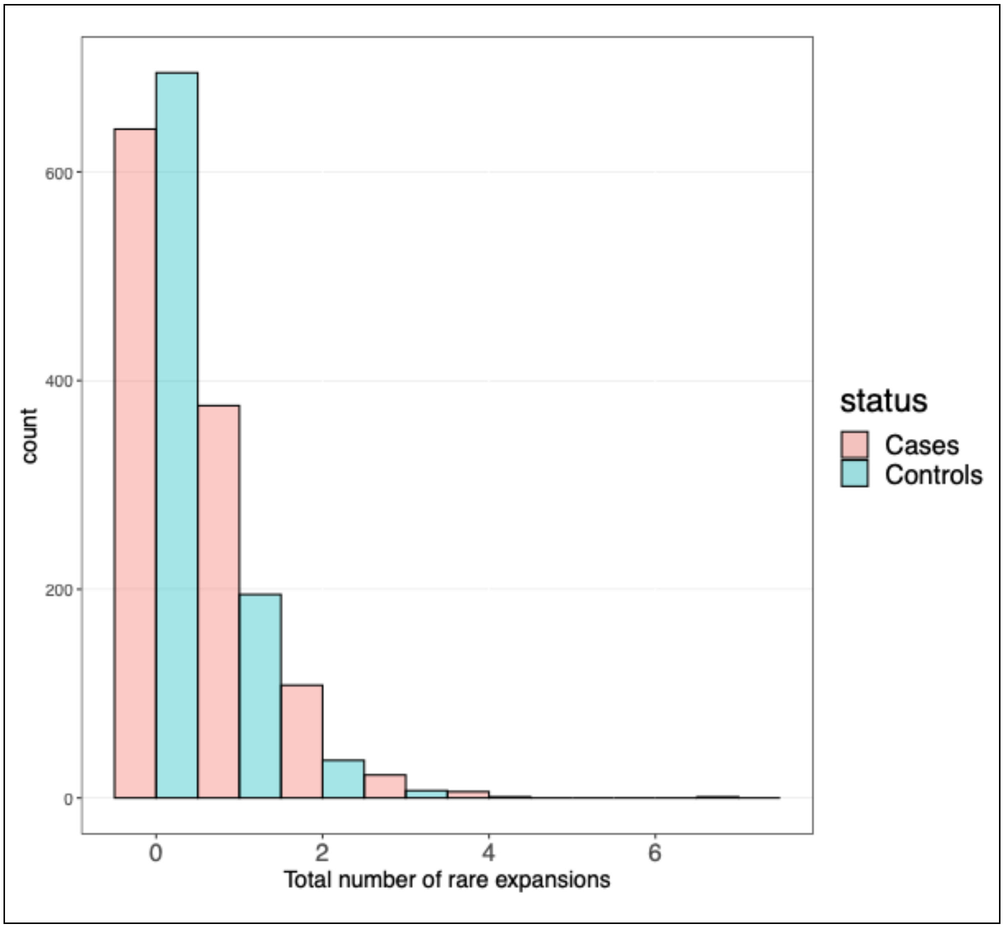

Supplementary Figure 8: Power calculation

Power calculation for burden tests was conducted using the R/gap package (v1.2.1). Our post-QC sample size is 1,154 schizophrenia cases and 936 controls. We assumed an additive model, lifetime risk of schizophrenia of 1%, type I error level of $5x10^{-8}$ (Supplementary Figure 4a), or $1x10^{-5}$ (Supplementary Figure 4b). We computed the minimal detectable genotypic relative risk to achieve 20%, 80% power over a range of frequency of risk alleles in the population. For burden tests, the X-axis of the power plots represents the aggregated frequency of a set of variants aggregated for a target region.

a. Assuming type I error level of $5x10^{-8}$

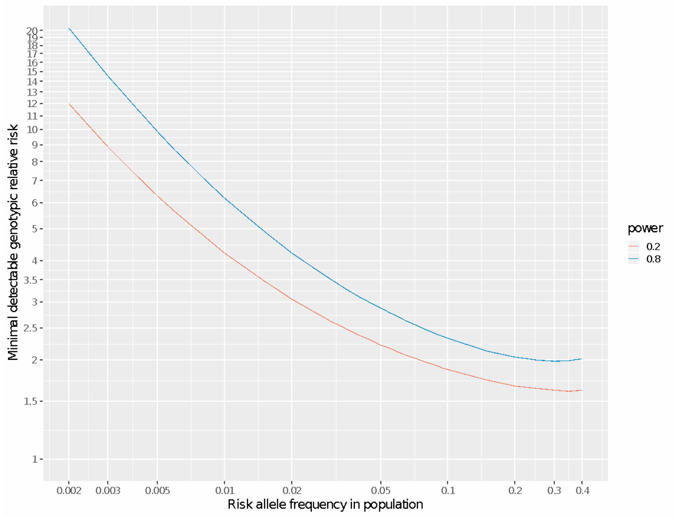

b. Assuming type I error level of $1x10^{-5}$

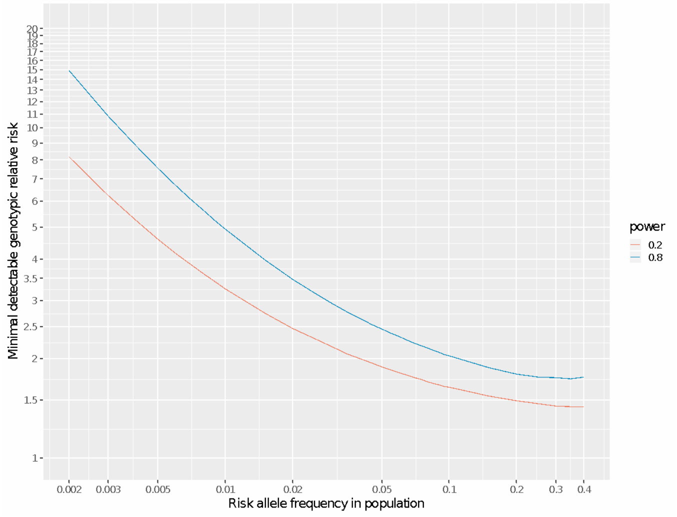

Supplementary Figure 9 Principal Component Analysis

In Supp Figure 9a, we performed a principal component analysis of the normalized anchored in-repeat reads (IRR) counts and plotted the second (PC2) vs the first PC (PC1). In Supp Figure 9b, we examined the effect of PC2 on the total number of tandem repeats identified per sample. Our results show that PC1 may represent the genetic signal we wish to detect while PC2 may confound the case control comparison in burden testing. We have corrected the confounding effect of PC2 in all subsequent burden tests by including it as a covariate in logistic regression models.

a.

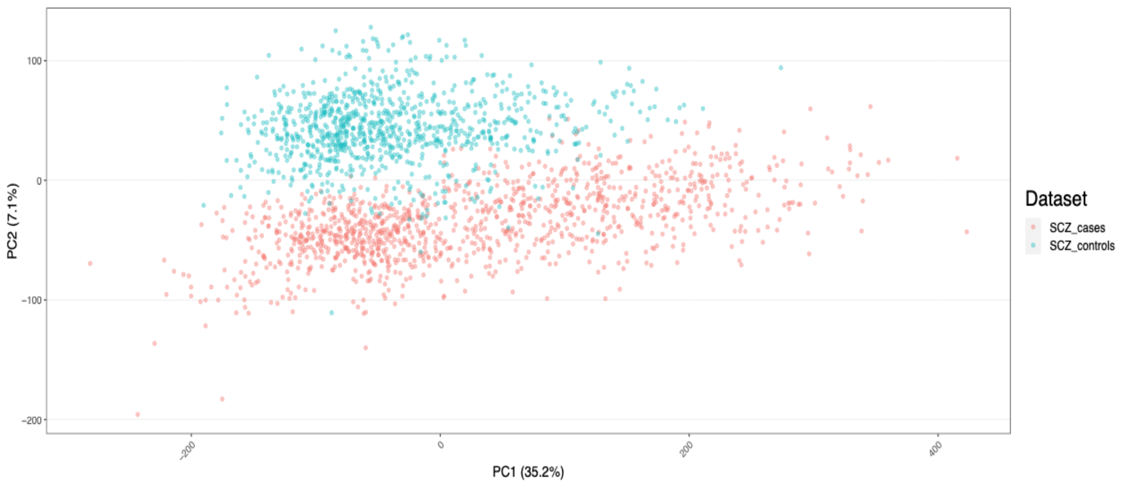

b.

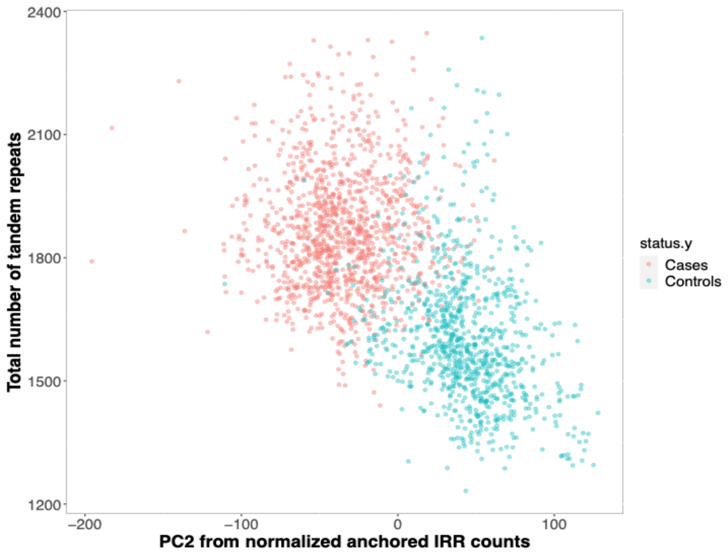

Supplementary Figure 10 IGV read pile-up in regions of interests

Integrative Genomics Viewer (IGV) read pile-up showing the reads aligning to the loci that had gel electrophoresis analysis. For each locus, we show IGV figures in two carriers (both schizophrenia cases) and one non-carrier for comparison. Color code: orange=G, blue=C, green=A, red=T. The coordinates in IGV plots were based on hg19 since bam files were based on hg19 and the coordinates listed in the caption were based on hg38.

Supplementary Figure 10.1 *PDIA5* chr3:123151382 -123152260 (AGGC)

a. SCZ case 1- TRE carrier

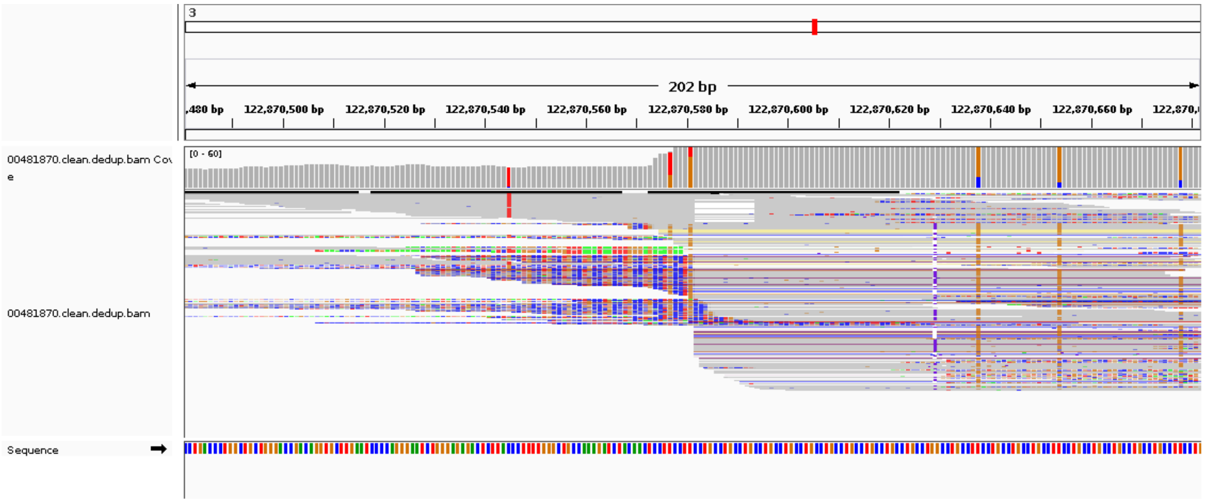

b. Non-carrier/control

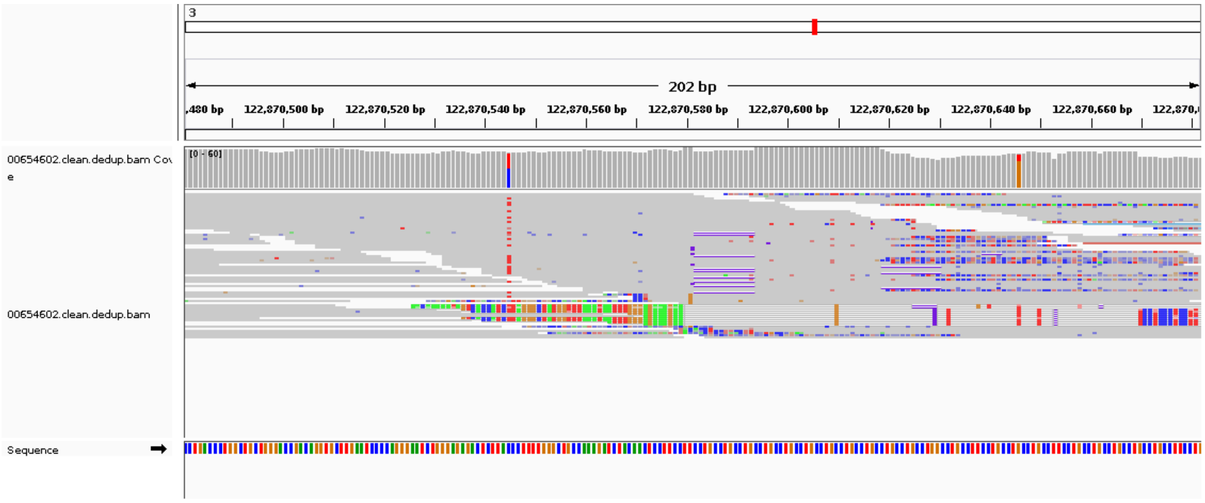

Supplementary Figure 10.2 *GABRA1* chr5:161663181-161664010 (AAATCATTT)

a. SCZ case 2 – TRE carrier

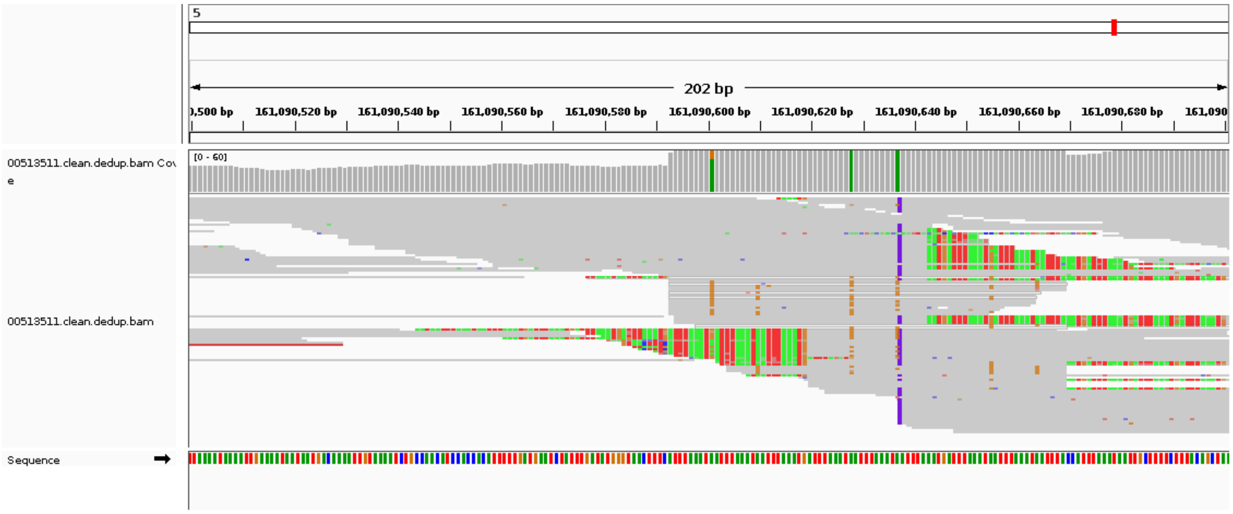

b. SCZ case 3 – TRE carrier

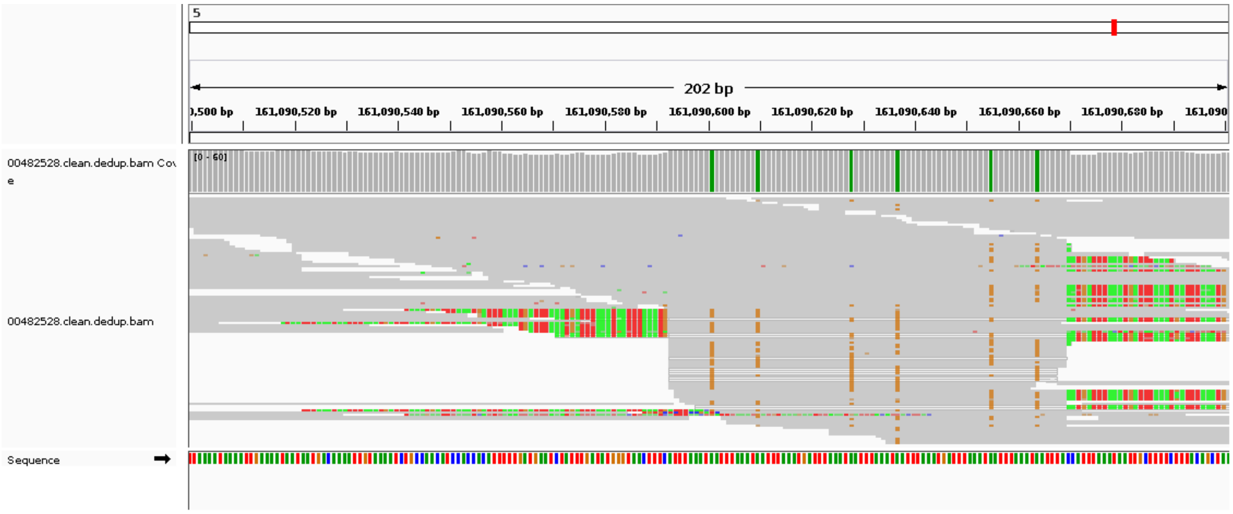

c. Non-carrier/control

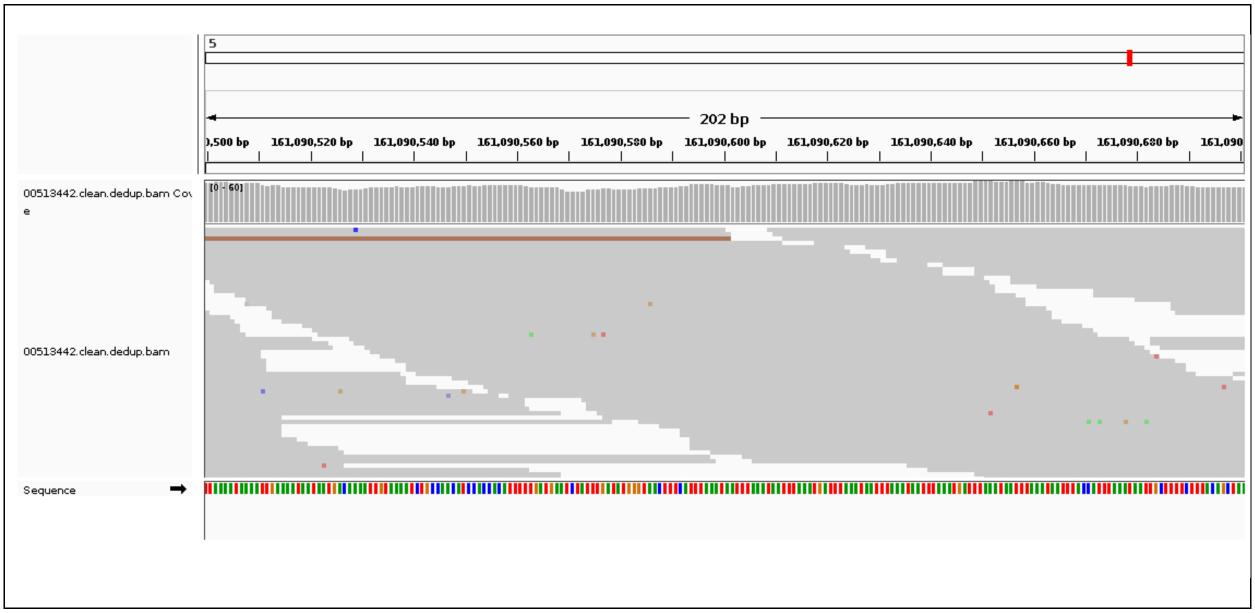
5
202 bp
),500 bp
161,090,520 bp
161,090,540 bp
161,090,560 bp
161,090,580 bp
161,090,600 bp
161,090,620 bp
161,090,640 bp
161,090,660 bp
161,090,680 bp
161,090
00513442.clean.dedup.bam Cov
e
[0 - 60]
00513442.clean.dedup.bam
Sequence

References

1. Dolzhenko E, Bennett MF, Richmond PA, Trost B, Chen S, van Vugt J *et al.* ExpansionHunter Denovo: a computational method for locating known and novel repeat expansions in short-read sequencing data. *Genome biology* 2020; **21**(1)**:** 102.
2. Halvorsen M, Huh R, Oskolkov N, Wen J, Netotea S, Giusti-Rodriguez P *et al.* Increased burden of ultra-rare structural variants localizing to boundaries of topologically associated domains in schizophrenia. *Nat Commun* 2020; **11**(1)**:** 1842.
3. Byrska-Bishop M, Evani US, Zhao X, Basile AO, Abel HJ, Regier AA *et al.* High coverage whole genome sequencing of the expanded 1000 Genomes Project cohort including 602 trios. *bioRxiv preprint doi: https://doiorg/101101/20210206430068* 2021.
4. Trost B, Engchuan W, Nguyen CM, Thiruvahindrapuram B, Dolzhenko E, Backstrom I *et al.* Genome-wide detection of tandem DNA repeats that are expanded in autism. *Nature* 2020; **586**(7827)**:** 80-86.
5. Zoonomia C. A comparative genomics multitool for scientific discovery and conservation. *Nature* 2020; **587**(7833)**:** 240-245.
6. Bryois J, Garrett ME, Song L, Safi A, Giusti-Rodriguez P, Johnson GD *et al.* Evaluation of chromatin accessibility in prefrontal cortex of individuals with schizophrenia. *Nat Commun* 2018; **9**(1)**:** 3121.
7. Consortium EP. An integrated encyclopedia of DNA elements in the human genome. *Nature* 2012; **489**(7414)**:** 57-74.
8. Giusti-Rodriguez PMD, Sullivan PF. Using three-dimensional regulatory chromatin interactions from adult and fetal cortex to interpret genetic results for psychiatric disorders and cognitive traits. *bioRxiv preprint doi: https://doiorg/101101/406330* 2019.
9. An L, Yang T, Yang J, Nuebler J, Xiang G, Hardison RC *et al.* OnTAD: hierarchical domain structure reveals the divergence of activity among TADs and boundaries. *Genome biology* 2019; **20**(1)**:** 282.
10. Girdhar K, Hoffman GE, Jiang Y, Brown L, Kundakovic M, Hauberg ME *et al.* Cell-specific histone modification maps in the human frontal lobe link schizophrenia risk to the neuronal epigenome. *Nat Neurosci* 2018; **21**(8)**:** 1126-1136.
11. Hu B, Won H, Mah W, Park RB, Kassim B, Spiess K *et al.* Neuronal and glial 3D chromatin architecture informs the cellular etiology of brain disorders. *Nat Commun* 2021; **12**(1)**:** 3968.
12. Fromer M, Roussos P, Sieberts SK, Johnson JS, Kavanagh DH, Perumal TM *et al.* Gene expression elucidates functional impact of polygenic risk for schizophrenia. *Nat Neurosci* 2016; **19**(11)**:** 1442-1453.
13. Lek M, Karczewski KJ, Minikel EV, Samocha KE, Banks E, Fennell T *et al.* Analysis of protein-coding genetic variation in 60,706 humans. *Nature* 2016; **536**(7616)**:** 285-291.
14. Cotney J, Muhle RA, Sanders SJ, Liu L, Willsey AJ, Niu W *et al.* The autism-associated chromatin modifier CHD8 regulates other autism risk genes during human neurodevelopment. *Nat Commun* 2015; **6:** 6404.
15. Darnell JC, Jensen KB, Jin P, Brown V, Warren ST, Darnell RB. Fragile X mental retardation protein targets G quartet mRNAs important for neuronal function. *Cell* 2001; **107**(4)**:** 489-499.
16. Singh T, Neale BM, Daly MJ. Exome sequencing identifies rare coding variants in 10 genes which confer substantial risk for schizophrenia. *medRxiv preprint doi: https://doiorg/101101/2020091820192815* 2020.
17. Marshall CR, Howrigan DP, Merico D, Thiruvahindrapuram B, Wu W, Greer DS *et al.* Contribution of copy number variants to schizophrenia from a genome-wide study of 41,321 subjects. *Nat Genet* 2017; **49**(1)**:** 27-35.

18. Trubetskoy V, Pardinas AF, Qi T, Panagiotaropoulou G, Awasthi S, Bigdeli TB *et al.* Mapping genomic loci implicates genes and synaptic biology in schizophrenia. *Nature* 2022.
19. Satterstrom FK, Kosmicki JA, Wang J, Breen MS, De Rubeis S, An JY *et al.* Large-Scale Exome Sequencing Study Implicates Both Developmental and Functional Changes in the Neurobiology of Autism. *Cell* 2020; **180**(3)**:** 568-584 e523.
20. Kaplanis J, Samocha KE, Wiel L, Zhang Z, Arvai KJ, Eberhardt RY *et al.* Integrating healthcare and research genetic data empowers the discovery of 49 novel developmental disorders. *bioRxiv preprint doi: https://doiorg/101101/797787* 2020.
21. Kochinke K, Zweier C, Nijhof B, Fenckova M, Cizek P, Honti F *et al.* Systematic Phenomics Analysis Deconvolutes Genes Mutated in Intellectual Disability into Biologically Coherent Modules. *American journal of human genetics* 2016; **98**(1)**:** 149-164.
22. Coe BP, Witherspoon K, Rosenfeld JA, van Bon BW, Vulto-van Silfhout AT, Bosco P *et al.* Refining analyses of copy number variation identifies specific genes associated with developmental delay. *Nat Genet* 2014; **46**(10)**:** 1063-1071.
23. Bragin E, Chatzimichali EA, Wright CF, Hurles ME, Firth HV, Bevan AP *et al.* DECIPHER: database for the interpretation of phenotype-linked plausibly pathogenic sequence and copy-number variation. *Nucleic acids research* 2014; **42**(Database issue)**:** D993-D1000.
24. Stefansson H, Meyer-Lindenberg A, Steinberg S, Magnusdottir B, Morgen K, Arnarsdottir S *et al.* CNVs conferring risk of autism or schizophrenia affect cognition in controls. *Nature* 2014; **505**(7483)**:** 361-366.
25. Wang D, Liu S, Warrell J, Won H, Shi X, Navarro FCP *et al.* Comprehensive functional genomic resource and integrative model for the human brain. *Science* 2018; **362**(6420).
26. Mojarad BA, Engchuan W, Trost B, Backstrom I, Yin Y, Thiruvahindrapuram B *et al.* Genome-wide tandem repeat expansions contribute to schizophrenia risk. *Mol Psychiatry* 2022.
27. Mojarad BA, Yin Y, Manshaei R, Backstrom I, Costain G, Heung T *et al.* Genome sequencing broadens the range of contributing variants with clinical implications in schizophrenia. *Transl Psychiatry* 2021; **11**(1)**:** 84.
28. Willer CJ, Li Y, Abecasis GR. METAL: fast and efficient meta-analysis of genomewide association scans. *Bioinformatics* 2010; **26**(17)**:** 2190-2191.
29. Karczewski KJ, Francioli LC, Tiao G, Cummings BB, Alföldi J, Wang Q *et al.* The mutational constraint spectrum quantified from variation in 141,456 humans. *Nature* 2020; **581**(7809)**:** 434-443.
30. Warde-Farley D, Donaldson SL, Comes O, Zuberi K, Badrawi R, Chao P *et al.* The GeneMANIA prediction server: biological network integration for gene prioritization and predicting gene function. *Nucleic acids research* 2010; **38**(Web Server issue)**:** W214-220.
31. Montojo J, Zuberi K, Rodriguez H, Kazi F, Wright G, Donaldson SL *et al.* GeneMANIA Cytoscape plugin: fast gene function predictions on the desktop. *Bioinformatics* 2010; **26**(22)**:** 2927-2928.
32. Shannon P, Markiel A, Ozier O, Baliga NS, Wang JT, Ramage D *et al.* Cytoscape: a software environment for integrated models of biomolecular interaction networks. *Genome research* 2003; **13**(11)**:** 2498-2504.
33. Werling DM, Brand H, An JY, Stone MR, Zhu L, Glessner JT *et al.* An analytical framework for whole-genome sequence association studies and its implications for autism spectrum disorder. *Nat Genet* 2018; **50**(5)**:** 727-736.

34. Siepel A, Bejerano G, Pedersen JS, Hinrichs AS, Hou M, Rosenbloom K *et al.* Evolutionarily conserved elements in vertebrate, insect, worm, and yeast genomes. *Genome research* 2005; **15**(8)**:** 1034-1050.
